# Ethanol production potential from AFEX™ and steam-exploded sugarcane residues for sugarcane biorefineries

**DOI:** 10.1186/s13068-018-1130-z

**Published:** 2018-05-04

**Authors:** Thapelo Mokomele, Leonardo da Costa Sousa, Venkatesh Balan, Eugéne van Rensburg, Bruce E. Dale, Johann F. Görgens

**Affiliations:** 1 Department of Process Engineering, Stellenbosch University, Private Bag X1 Matieland, Stellenbosch, South Africa; 2 Biomass Conversion Research Laboratory, Department of Chemical Engineering and Materials Science, Michigan State University, East Lansing, USA; 3 Great Lakes Bioenergy Research Center (GLBRC), Michigan State University, East Lansing, MI USA; 4 Department of Engineering Technology, Biotechnology Program, School of Technology, University of Houston, 4800 Calhoun, Road, Houston, TX 77004 USA

**Keywords:** AFEX™, Bagasse, Cane leaf matter, Enzymatic hydrolysis, Ethanol yield, Fermentation, Sugarcane cultivation area, Steam explosion

## Abstract

**Background:**

Expanding biofuel markets are challenged by the need to meet future biofuel demands and mitigate greenhouse gas emissions, while using domestically available feedstock sustainably. In the context of the sugar industry, exploiting under-utilized cane leaf matter (CLM) in addition to surplus sugarcane bagasse as supplementary feedstock for second-generation ethanol production has the potential to improve bioenergy yields per unit land. In this study, the ethanol yields and processing bottlenecks of ammonia fibre expansion (AFEX™) and steam explosion (StEx) as adopted technologies for pretreating sugarcane bagasse and CLM were experimentally measured and compared for the first time.

**Results:**

Ethanol yields between 249 and 256 kg Mg^−1^ raw dry biomass (RDM) were obtained with AFEX™-pretreated sugarcane bagasse and CLM after high solids loading enzymatic hydrolysis and fermentation. In contrast, StEx-pretreated sugarcane bagasse and CLM resulted in substantially lower ethanol yields that ranged between 162 and 203 kg Mg^−1^ RDM. The ethanol yields from StEx-treated sugarcane residues were limited by the aggregated effect of sugar degradation during pretreatment, enzyme inhibition during enzymatic hydrolysis and microbial inhibition of *S. cerevisiae* 424A (LNH-ST) during fermentation. However, relatively high enzyme dosages (> 20 mg g^−1^ glucan) were required irrespective of pretreatment method to reach 75% carbohydrate conversion, even when optimal combinations of Cellic^®^ CTec3, Cellic^®^ HTec3 and Pectinex Ultra-SP were used. Ethanol yields per hectare sugarcane cultivation area were estimated at 4496 and 3416 L ha^−1^ for biorefineries using AFEX™- or StEx-treated sugarcane residues, respectively.

**Conclusions:**

AFEX™ proved to be a more effective pretreatment method for sugarcane residues relative to StEx due to the higher fermentable sugar recovery and enzymatic hydrolysate fermentability after high solids loading enzymatic hydrolysis and fermentation by *S. cerevisiae* 424A (LNH-ST). The identification of auxiliary enzyme activities, adequate process integration and the use of robust xylose-fermenting ethanologens were identified as opportunities to further improve ethanol yields from AFEX™- and StEx-treated sugarcane residues.

**Electronic supplementary material:**

The online version of this article (10.1186/s13068-018-1130-z) contains supplementary material, which is available to authorized users.

## Background

Sustainably produced liquid biofuels are key to a projected future where biomass-derived biofuels will partially displace petroleum-based transportation fuels [1]. The progressive transition toward indigenous cellulosic second-generation (2G) biofuel production from first generation (1G), which uses food resources, can potentially facilitate environmental, economic and socio-economic benefits in both developing and developed countries [2, 3]. While 2G biofuel technology is steadily entering the commercial deployment phase, major impediments to its commercial appeal remain, specifically related to the feedstock supply chain, land availability for expansion, technology maturity and overall economic feasibility [2, 4, 5].

Sugarcane is a major agricultural crop widely considered as one of the leading candidates for bioenergy, with Brazil producing 691 million tons of sugarcane during the 2016–2017 harvest season [6]. First-generation ethanol produced from sugarcane (from extractable sugars) is a commercial process with an industrial maturity of greater than 40 years [7]. However, with a growing world population and biofuel demand, expanding biofuel production beyond existing farmlands is challenged by land conservation concerns, especially in countries with limited capacity for sugarcane cultivation area expansion [8–10]. Consequently, there is substantial interest in crop variety selection and the utilization of the whole sugarcane plant for biofuel production as sustainable approaches to increasing sugarcane ethanol yields per unit land [11].

The sugarcane processing industry typically generates approximately 140 kg dry weight bagasse (fibrous residue after juice extraction) and an equal amount (dry weight) of cane leaf matter (green leaves, tops and trash) per ton of wet harvested cane [12]. Presently, bagasse is burned in inefficient mill boilers to produce heat and electricity for sugar milling operations, with surplus energy exported to the grid [13, 14]. Improvements in the sugar mill operation energy efficiency and investment in more energy-efficient power cogeneration technology would liberate surplus bagasse for future biorefinery applications [13, 15]. Moreover, it has previously been common practice to burn sugarcane cane leaf matter (CLM) on the stalk prior to harvesting to facilitate easier and cheaper sugarcane stalk collection and transportation [13, 16, 17]. As a result of environmental regulations coupled with an industry-wide effort to phase out CLM burning, the utilization of this biomass as substrate for bioconversion to bioethanol, electricity and/or other value-added products in a biorefinery setting provides an alternative, potentially greener and more sustainable approach [18]. Whereas the requirements for sustainable agriculture prevent the complete removal of CLM from the field due to reduced soil fertility over a period of years, some studies suggest that 50% of the sugarcane harvest residues can be removed from the field, with the remainder ploughed back in to soil without significantly affecting nutrient cycling, soil biodiversity, soil carbon sequestration and pest control [18–21]. Therefore, depending on the amount of CLM that can be recovered from the field and proximity to the sugar mill, these residues can either be baled or transported together with the sugarcane stalk to the sugar mill to supply either 2G biofuel production or energy cogeneration [22]. The availability of these residues as either supplementary feedstock to sugarcane juice in integrated 1G–2G biorefineries or as sole feedstock in standalone 2G biorefineries annexed to sugar mills, has the potential to enhance the ethanol yield per unit land without expanding the cultivation area, while maximizing environmental benefits and minimizing capital and production costs [15, 20, 23]. In addition to energy integration benefits, these 2G sugarcane residue biorefineries integrated to sugar mills or 1G biorefineries present an attractive opportunity for sharing of existing feedstock supply, handling infrastructure and logistical systems that currently represent a significant hurdle for the nascent 2G biofuel production industry [24].

To compete with traditional petroleum refineries, high biomass-to-biofuel yields with low enzyme loadings are required for the biochemical processing of recalcitrant sugarcane residues [25, 26]. Although there are numerous pretreatment technologies with different biomass deconstruction chemistries, most pretreatments present various economic and environmental challenges concerning costly chemical use and recovery, excess water use, feedstock handling, energy requirements and downstream solids processing [25]. Among the leading thermochemical pretreatment options, steam explosion (StEx) and ammonia fibre expansion (AFEX™) are two well-studied and scalable technologies (demonstrated at pilot scale) that are being considered for overcoming biomass recalcitrance, given their different biomass deconstruction patterns (acidic vs alkaline) and potential for integration into existing sugarcane mills [27, 28].

Autocatalyzed StEx is a well-known thermochemical pretreatment approach that uses high-temperature saturated steam and intrinsic biomass-derived organic acids (e.g. acetic acid) to enhance cellulose digestibility. During the pretreatment process, there is selective fractionation of hemicellulose, partial cleavage of lignin–carbohydrate complex ester linkages and increased substrate accessibility toward hydrolytic enzymes [29–32]. Advantages of StEx pretreatment for integration in sugar mill operations include the use of water as a green solvent, relatively low capital investment, moderate energy requirements and the ability to use high-moisture content biomass (such as bagasse) [31, 33]. However, due to pretreatment severities required for obtaining high cellulose digestibility, StEx generates hemicellulose and cellulose-derived degradation products that are inhibitory to downstream enzymatic hydrolysis and fermentation [34]. To avoid limiting biomass-to-biofuel yields due to the presence of inhibitory compounds, the pretreatment slurry has been previously separated by means of a solid–liquid separation step followed by washing the residual solid with water to remove soluble sugars and inhibitors [35]. However, during commercial application, it is likely that either unwashed (pressed) solids or whole slurries (hydrolysate liquor plus solids) will be preferred in view of minimizing process water consumption and downstream water recovery costs [30, 36, 37]. Therefore, detailed carbohydrate-to-biofuel yields are necessary to understand the benefits of washing/separating the pretreatment slurry to mitigate the impact of pretreatment-derived inhibitors on enzymatic hydrolysis and microbial fermentation.

In comparison, an alkaline pretreatment process, AFEX™ (trademark of MBI International, Lansing, Michigan) treats moist biomass with anhydrous ammonia at moderate temperatures and pressures, followed by the rapid release of pressure and recovery of vaporized ammonia [38]. AFEX™ is a “dry-to-dry” process that eliminates the requirements for wastewater recovery and solid–liquid separations. Recent advances in renewable hydrogen production and the subsequent production of ammonia from renewable hydrogen provide enthusiasm for the future use of ammonia as a green solvent [39]. AFEX™ pretreatment enhances biomass enzymatic digestibility through the cleavage of lignin–carbohydrate complex ester linkages, cellulose de-crystallization, de-acetylation, lignin/hemicellulose redistribution towards the outer plant cell wall, and increased enzyme-accessible area. Furthermore, AFEX™ preserves the native plant nutrients and generates minimal inhibitory degradation products, resulting in a fermentable enzymatic hydrolysate that does not require detoxification or significant external nutrient supplementation [40]. However, ammonia recovery operations and make-up ammonia increase the capital and operating costs for AFEX™. Therefore, optimizing pretreatment conditions at low ammonia to biomass loading has been proposed as a potential strategy to reducing ammonia recovery costs [41].

In this study, the potential ethanol yields that can be recovered from StEx- and AFEX™-treated sugarcane bagasse and CLM at industrially relevant conditions were explored and compared for the first time. A wide range of StEx and AFEX™ pretreatment conditions were evaluated for sugarcane bagasse and CLM, followed by selecting conditions that facilitate high sugar recovery at moderate enzyme loading with limited pretreatment catalyst loading. To establish the effect of solids separation and/or washing, high solids loading enzymatic hydrolysis experiments were performed at varying enzyme loadings using optimized combinations of Cellic^®^ CTec3, Cellic^®^ HTec3 and Pectinex Ultra-SP. Further, the fermentability of all carbohydrate fractions from both AFEX™ and StEx were evaluated to determine the extent of microbial inhibition due to AFEX™- and StEx-pretreatment-derived inhibitors. From carbohydrate and ethanol mass balances, the potential ethanol yields per unit land for sugarcane biorefineries based on either AFEX™ or StEx for 2G ethanol production were estimated. Ultimately, this work provides data and insights that will enable subsequent economic evaluations of the various processing options for future StEx- or AFEX™-based sugarcane residue ethanol biorefineries.

## Methods

### Biomass collection and preparation

Sugarcane bagasse (at 50–60% w/w moisture content) and manually harvested cane leaf matter (including green leaves, tops and trash) were collected from two sugarcane mills located in Malelane (TSB Sugar, Mpumalanga) and Mount Edgecombe (SASRI, Kwazulu Natal), South Africa. To prevent biomass spoilage, the bagasse and CLM were air-dried in separate greenhouses until the equilibrium moisture content was approximately 7% (w/w). The bagasse was milled using a laboratory toothed disk mill (Condux LV15M, Netzch-Condux GmbH, Germany) and passed through a 20 mm screen. The size-reduced bagasse samples were sieved in a stacked-sieve system to remove mineral impurities (e.g. sand), bagasse pith and fines that are smaller than 600 μm × 600 μm. De-pithing bagasse is common practice in South African sugar mills to facilitate the use of longer bagasse fibres as fuel for steam/energy production, and the bagasse pith is typically used as a molasses carrier in animal feed products [42]. The bagasse from two sources was thoroughly mixed and stored in vacuum-sealed bags at room temperature until use.

Air-dried CLM was hammer-milled (Massey-Ferguson, USA) and passed through a hexagonal screen with a 20 mm diameter to attain particles with an approximate length ranging between 50 and 70 mm. The milled CLM samples were sieved to remove mineral impurities and fines smaller than 600 μm × 600 μm. The CLM from both sources was well mixed to achieve a representative sample of South African post-harvest CLM and stored in vacuum-sealed bags at room temperature until use.

### Composition analysis

The composition of the raw biomass samples was determined according to National Renewable Energy Laboratory (NREL) protocols NREL/TP-510-42618 and NREL/TP-510-42620. The higher heating value (HHV) was measured using a bomb calorimeter (Cal2 k Eco Calorimeter) based on ASTM standard D5865-11a. Statistical significance between experimental values was determined through the application of a one-way ANOVA in combination with Tukey’s HSD post hoc test for multiple comparisons (Minitab Inc., State College, PA, USA). A *p* value less than 0.05 was considered statistically significant.

### Steam explosion

Steam explosion (StEx) was performed in an automated batch pilot scale unit (IAP GmBH, Graz, Austria) equipped with a 19 L reaction vessel, a 100 L expansion vessel and a 40 bar steam boiler [43]. In preparation for StEx pretreatment, untreated sugarcane bagasse or CLM was pre-soaked in reverse-osmosis water overnight at a solid-to-water ratio of 1:2 to ensure maximum water absorption into the biomass. The water-impregnated material was subsequently dewatered in a gravity drain spin dryer (AEG SV4028, Germany) to a moisture content akin to industrial bagasse (65–75%). The StEx reaction vessel, preheated to 185 °C, was top-loaded with 500 g (dry basis) of water-impregnated bagasse or CLM and directly heated to the desired temperature using 30 bar (absolute) saturated. After the required pretreatment time had elapsed, the reactor contents were discharged into the expansion vessel maintained at atmospheric pressure. Each pretreatment was performed in duplicate. Three 100 g samples of the pretreatment slurry were characterized in terms of the total solids (TS), water-soluble solids (WSS), water-insoluble solids (WIS), and pH. The remaining slurry was separated into a solid (pressed solids) and a liquid fraction (pretreatment C_5_-liquor) using a pneumatic piston press (Eurotool TY5001, South Africa). The pressed (unwashed) solids with an approximate moisture content of 65% (w/w) were air-dried at 35 °C to a moisture content of 15% (w/w). The combined sugar yield for StEx was calculated from the soluble monomeric and oligomeric sugars (glucose + xylose) in the pretreatment liquor and the soluble monomeric sugars (glucose + xylose) released after low solids loading enzymatic hydrolysis (described below) of washed solids.

The bagasse and the CLM were pretreated at temperatures and residence times ranging from 185 to 215 °C and 10 to 15 min, respectively (Additional file 1: Table S1). For each biomass material, three pretreatment conditions were considered based on previous work and preliminary data from unpublished work by Hamann et al. [49–53]. First, low severity pretreatment conditions leading to high hemicellulose solubilization and recovery in the pretreatment liquor with low degradation product generation were evaluated. Secondly, high severity pretreatment conditions facilitating high cellulose digestibility in the pretreated fibres were evaluated. Lastly, intermediate severity pretreatment conditions resulting in high total sugar recovery from both the pretreatment liquor and enzymatic hydrolysis steps were evaluated.

### AFEX™ pretreatment

#### High-throughput batch AFEX™

High-throughput AFEX™ pretreatment was performed in 22-mL pressure vessels (Parr Instrument Company, Moline, IL, USA) [44]. To facilitate the high-throughput pretreatments, untreated sugarcane bagasse and CLM samples were milled and passed through a 5-mm screen using a Wiley Mill. AFEX™ conditions for evaluating the effect of pretreatment conditions were selected using a central composite statistical design (CCD) (Additional file 2: Table S2). Experimental data were taken within ammonia loading, water loading and pretreatment temperature ranges between 0.5 and 1.5 g NH_3_/g dry biomass, 0.4 and 0.8 g H_2_O/g dry biomass, and 100 and 140 °C, respectively. A minimum of 40 experimental data points was generated for statistical analysis using Minitab software (Minitab Inc., State College, PA, USA) for sugarcane bagasse and CLM each, including duplicates and five centre point replicates. The combined sugar yield (monomeric glucose + xylose) from low solids loading enzymatic hydrolysis (see below) was used as the metric of pretreatment efficacy. A full quadratic model was used to fit the experimental data containing all three pretreatment variables, including their main, interaction and quadratic effects. The models were refined to include parameters deemed significant by ANOVA and influence of the model predictive ability (*p *< 0.05 and *R*^2^_predicted_). The regression models were validated and used to predict the effect of the pretreatment conditions on the sugar yield within the experimental boundaries.

#### Pre-pilot scale AFEX™

Pre-pilot scale AFEX™ pretreatment was performed in a 3.8 L high-pressure reaction vessel (Parr) equipped with temperature and pressure sensors, as described previously [45]. Sugarcane bagasse was treated with AFEX™ at 0.6 g H_2_O/g dry biomass, and 1.0 g NH_3_/g dry biomass, 140 ± 2 °C, and 60 min. AFEX™ treatment of CLM was performed at 0.7 g H_2_O/g dry biomass, and 1.0 g NH_3_/g dry biomass, 135 ± 2 °C, and 30 min. Each pretreatment was performed in duplicate. Pretreated samples were stored in sealed bags at 4 °C prior to enzymatic hydrolysis at low and high solids loading.

### Enzymes

Commercial fungal enzyme preparations Cellic^®^ CTec2 and Cellic^®^ HTec2 were used to determine the effect of StEx pretreatment conditions and were generously donated by Novozymes (Copenhagen, Denmark). Commercially relevant Cellic^®^ CTec3, Cellic^®^ HTec3 and Pectinex Ultra-SP were used in subsequent studies with AFEX™ pretreatment optimization, enzyme mixture optimization and high solids loading enzymatic hydrolysis. These preparations were also generously donated by Novozymes Inc. (Franklinton, NC, USA). The protein concentration of the enzyme preparations was estimated using Kjeldahl nitrogen analysis (AOAC Method 2001.11, Dairy One Corporative Inc., Ithaca, NY, USA).

### Low solids loading enzymatic hydrolysis

Low solids loading enzymatic hydrolysis was used to determine the impact of AFEX™ and StEx pretreatments on the sugar release from the pretreated solids. After StEx pretreatment, enzymatic hydrolysis was performed at a solids loading of 2% (w/v) WIS in 100-mL shake flasks at a total enzyme dosage of 33 mg protein g^−1^ glucan and incubated at 50 °C, and pH 4.8 for 72 h on an orbital shaker (Lasec SA, Cape Town, South Africa) adjusted to 150 rpm. A fixed enzyme cocktail mixture consisting of 22 mg CTec2/g glucan and 11 mg HTec2/g glucan was used. The reaction mixture was supplemented with 50 mM citrate buffer and 0.02% (w/v) sodium azide (Sigma Aldrich, South Africa) to maintain the hydrolysis pH and to prevent microbial contamination, respectively.

During the optimization of AFEX™ pretreatment, enzymatic hydrolysis was performed in 20-mL screw-cap scintillation vials at 1% (w/v) glucan loading using 15 mg protein g^−1^ of glucan, incubated at 50 °C, pH 4.8 for 72 h in an orbital shaker (New Brunswick Scientific, Edison, NJ, USA). A standard enzyme cocktail mixture consisting of 10 mg CTec3/g glucan and 5 mg HTec3/g glucan was used. After enzymatic hydrolysis, samples of the hydrolysate were withdrawn, incubated at 95 °C for 20 min (Thermomixer^®^ R, Eppendorf, Westbury, USA) to denature the enzymes, and prepared for HPLC analysis.

### Enzyme mixture optimization

A second-degree simplex lattice mixture design was carried out to determine optimal combinations of commercial enzymes Cellic^®^ CTec3, Cellic^®^ HTec3 and Pectinex Ultra-SP for the release of sugars from optimally pretreated AFEX™ and StEx sugarcane bagasse and CLM. The total enzyme dosage was fixed at 15 mg total protein/g glucan and the ratio of the enzymes ranged from 0 to 1. A total of 40 experiments were generated in Minitab software for each pretreated substrate, including replicates (Minitab Inc.). The monomeric combined sugar yield (glucose + xylose) from low solids loading enzymatic hydrolysis was used to evaluate the effect of the different enzyme mixtures. Refined cubic regression models were generated, validated and used to predict the optimum enzyme combinations based on the combined sugar yield.

### High solids loading enzymatic hydrolysis

High solids loading enzymatic hydrolysis was performed in 250-mL baffled Erlenmeyer flasks with a 100 mL working volume, incubated at 50 °C, and pH 5.0 on an orbital shaker adjusted to 250 rpm (New Brunswick Scientific, NJ, USA). Enzymatic hydrolysis was performed at 10% (w/w) carbohydrate loading, defined as the sum of the insoluble glucan and xylan, soluble xylo-oligosaccharides (X-OS) and gluco-oligosaccharides (G-OS), and soluble monomeric glucose and xylose in the pretreated material. The enzymatic hydrolysis mixtures were supplemented with 50 mM phosphate buffer and 50 mg L^−1^ chloramphenicol to maintain the hydrolysis pH and prevent bacterial contamination, respectively. Optimized ratios of Cellic^®^ CTec3, Cellic^®^ HTec3 and Pectinex Ultra-SP were used for the various pretreated feedstocks at enzyme dosages that ranged between 7.5 and 45 mg enzyme g^−1^ glucan.

The hydrolysis was carried out using a fed-batch strategy in which half the biomass added at *t* = 0 h, and the remainder added at *t* = 3 h. After a 96-h hydrolysis period, the slurry was centrifuged at 10,000*×g* for 30 min to separate the unhydrolyzed solids from the hydrolysate. Samples of the hydrolysate were removed and analysed for monomeric and oligomeric sugar content. The unhydrolyzed solids were washed with 100 mL distilled water, centrifuged for a further 30 min at 10,000*×g*. The supernatant was analysed for sugar content for mass balance closure. In preparation for fermentation, the hydrolysate was supplemented with 0.25% (w/w) corn steep liquor, and the pH adjusted to 5.5 before being filter sterilized through a 0.22-µm filter and refrigerated at 4 °C until use.

### StEx C_5_-liquor post-hydrolysis treatment

Post-hydrolysis treatment with dilute sulphuric acid was performed to recover the oligomeric sugars in the StEx pretreatment hemicellulose-rich liquor (referred to as C_5_-liquor) in monomeric form. The hydrolysis was performed in 100-mL glass pressure tubes with Teflon caps and *o*-ring seals (Ace Glass, New Jersey, USA). About 80 mL of C_5_-liquor was added to the pressure tubes followed by the addition of 72% H_2_SO_4_ to achieve acid loadings of 1.0% (w/w) and 0.75% (w/w) for bagasse and CLM, respectively. The pressure tubes were autoclaved at 121 °C for 60 min and subsequently cooled in ice. After cooling, the liquor pH was adjusted to pH 5.0 using a 30% (v/v) ammonium hydroxide solution, supplemented with 0.25% (w/w) corn steep liquor, then re-adjusted to pH 5.5. The pH-adjusted C_5_-liquor was filter sterilized through a 0.22-µm filter and stored at 4 °C until use. Triplicate samples were prepared for each C_5_-liquor sample evaluated.

### Fermentation

The genetically modified, xylose-fermenting *Saccharomyces cerevisiae* strain 424A (LNH-ST), kindly provided by Prof. Nancy W.Y. Ho, Purdue University, was used to ferment AFEX™ and StEx enzymatic hydrolysates and the StEx C_5_-liquor. The seed culture of this strain was prepared in 250-mL Erlenmeyer flasks containing YPDX medium that consisted of (per litre) 75 g glucose, 25 g xylose, 10 g yeast extract and 20 g tryptone. A frozen glycerol stock was used for seed culture inoculation at an initial optical density of 0.1. The seed culture was cultivated at 30 °C and 150 rpm for 18 h to an approximate optical density (OD_600_) of 12. The culture was subsequently harvested and used as inoculum for AFEX™, StEx (washed solids) and StEx (pressed or unwashed solids) enzymatic hydrolysate fermentations. In experiments where the whole slurry after StEx pretreatment or StEx C_5_-liquor was fermented, the yeast was pre-conditioned in an additional cultivation step prior to inoculating the growth medium. Pre-conditioning was carried out by inoculating 75 mL YPDX media and 25 mL of C_5_-liquor in a 250-mL Erlenmeyer flask using the seed culture described above. After inoculating the pre-conditioning medium to an initial OD_600_ of 2, cultures were incubated in a rotary incubator adjusted to 30 °C and 150 rpm for 18 h. The pre-conditioned seed culture medium was centrifuged at 4000 rpm for 15 min and the yeast pellets were used as inoculum for StEx-whole slurry or C_5_-liquor fermentation.

Enzymatic hydrolyses and C_5_-liquor fermentations were performed in 125-mL Erlenmeyer flasks with 50 mL working volume at pH 5.5, 30 °C and 150 rpm for 120 h. A rubber stopper with a hypodermic needle piercing was used to cap the flask and maintain predominantly anaerobic conditions. The fermentation flasks were inoculated at OD_600_ of 2, which corresponded to a yeast biomass concentration of 0.96 g CDW L^−1^. Samples were withdrawn at frequent intervals and after centrifugation, the cell-free supernatants were prepared for HPLC analysis. The ethanol metabolic yield was calculated from the glucose and xylose consumed relative to the theoretical ethanol yield of 0.51 g ethanol g^−1^ glucose or xylose consumed. The overall process ethanol yield was determined based on the sugar yield from enzymatic hydrolysis and the sugar consumption and metabolic yield during fermentation. Monomeric sugars (glucose, xylose, arabinose), pretreatment products (acetic acid, formic acid) and fermentation products (lactate, xylitol, glycerol and ethanol) were determined by HPLC system equipped with an Aminex HPX-87H column (Bio-Rad, Hercules, CA, USA) as described previously [43]. The column temperature was maintained at 50 °C, with sulphuric acid (5 mM) used as the mobile phase at a flowrate of 0.6 mL min^−1^.

### Process configurations

**Fig. 1:**
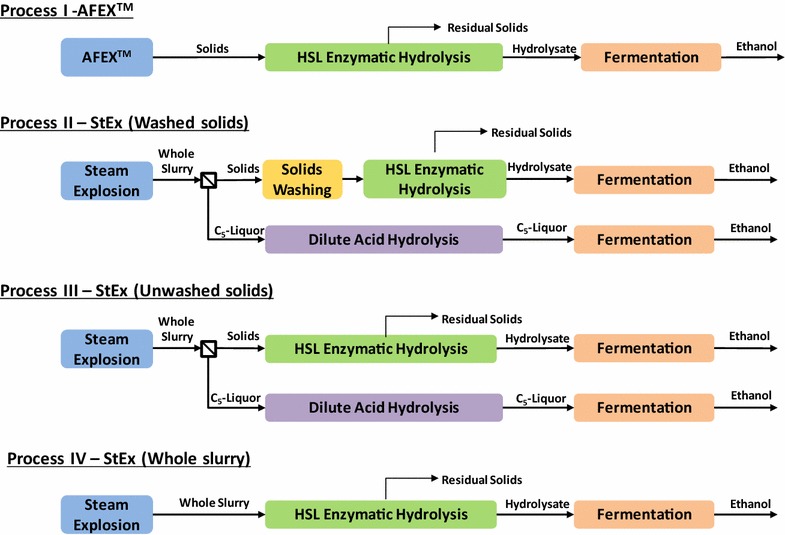
Process flowsheets studied for the conversion of sugarcane residues to ethanol. Process I—AFEX™ pretreatment with high solids loading separate enzymatic hydrolysis and fermentation (SHF) of the solids, Process II—Steam explosion followed by solids washing and high solids loading SHF with separate fermentation of the C_5_-rich liquor, Process III—steam explosion followed by high solids loading SHF of unwashed solids and separate fermentation of the C_5_-rich liquor, Process IV—steam explosion followed by high solids loading SHF of the whole slurry. *HSL* high solids loading

Four process configurations were evaluated for the pretreated materials using a separate hydrolysis and fermentation (SHF) flow scheme (Fig. 1). In Process I, AFEX™-treated bagasse or CLM underwent high solids loading enzymatic hydrolysis, followed by the removal of undigested solids and fermentation of the enzymatic hydrolysate. To determine the extent of enzymatic and microbial inhibition due to the presence of StEx-derived degradation products, the StEx-pretreated slurry was processed in three ways, referred to as Processes II, III and IV. In Process II, StEx pretreatment was followed by solid–liquid separation to recover the C_5_-rich liquor and the solid fraction. The solid fraction was washed in three stages with distilled water heated to 50 °C, using a total of 10 L water kg^−1^ pressed solids to remove soluble sugars and pretreatment-generated organic acids, furan derivatives and water-soluble phenolic compounds [46]. After washing, the solids were subjected to high solids loading enzymatic hydrolysis followed by separate fermentation of the enzymatic hydrolysate and the acid-hydrolysed C_5_-liquor. Lignin-rich residual solids were recovered after enzymatic hydrolysis. Process III was performed under identical conditions as Process II, except that the solids washing step was excluded. Finally, in Process IV, eliminating the solid/liquid separation and washing steps after StEx pretreatment was also evaluated, resulting in a one-stream, whole slurry configuration. Monomeric, oligomeric and polymeric sugars and ethanol concentrations before and after each process unit operation were determined and mass balances were calculated as previously described [40].

## Results and discussion

### Biomass composition and energy value

The composition and calorific value of sugarcane bagasse and CLM are presented in Table 1 and was similar to that previously reported for South African industrial sugarcane residues [47]. Sugarcane bagasse demonstrated higher glucan, acetyl group and lignin contents and lower extractives and ash contents relative to the CLM (*p* < 0.05). Based on the glucan and xylan contents, the potential monomeric sugar (glucose + xylose) recovery from of bagasse and CLM is 72.48 ± 0.6 and 69.75 ± 0.9 kg/100 kg RDM, respectively, making both materials promising feedstocks for ethanol production. The lower ash content and higher HHV of the bagasse (*p *< 0.05) suggest that it may be a more suitable source candidate for cogeneration operations in common mill boilers [17]. High ash content boiler feeds are understood to contribute to slagging, corrosion and fouling formation within the boiler [16, 48]. Other than washing the CLM to remove mineral impurities collected from harvesting the CLM, mixing with bagasse (at appropriate ratios) may provide a simpler way of reducing the ash content of sugar mill boiler feeds. Moreover, given the availability of sugarcane bagasse at elevated moisture levels (> 50%, w/w) as an end-of-process product compared to the modest moisture content of on-field dried CLM (∼ 15%, w/w), mixing the two feedstocks may also be an effective strategy of reducing the moisture content and increasing the efficiency of sugarcane mill boiler feeds.

**Table 1:** Chemical composition and energy value of sugarcane bagasse, cane leaf matter and a bagasse-CLM mixture

Biomass component^a^	Bagasse	Cane leaf matter	Bagasse + CLM mixture (1:1 w/w)
Glucan (kg/100 kg DM)	39.50 ± 0.41^A^	37.45 ± 0.6^B^	38.11 ± 0.14^B^
Xylan (kg/100 kg DM)	25.21 ± 0.13^A^	24.81 ± 0.4^A^	24.21 ± 0.2^B^
Arabinan (kg/100 kg DM)	1.23 ± 0.38^B^	2.73 ± 0.1^A^	1.48 ± 0.24^B^
Acetyl (kg/100 kg DM)	3.43 ± 0.04^B^	2.21 ± 0.06^C^	4.32 ± 0.18^A^
Lignin (kg/100 kg DM)	19.35 ± 0.06^A^	16.17 ± 0.81^B^	19.5 ± 0.59^A^
Ash (kg/100 kg DM)	2.89 ± 0.65^C^	7.34 ± 0.21^A^	5.21 ± 0.71^B^
Extractives (kg/100 kg DM)	6.02 ± 0.42^C^	12.07 ± 1.54^A^	10.32 ± 0.39^B^
Calorific value^a^			
Higher heating value (MJ kg^−1^)	18.47 ± 0.06^A^	17.67 ± 0.05^C^	17.92 ± 0.13^B^

Different superscripts within row indicate significant differences as determined using one-way ANOVA with Tukey’s HSD post hoc test for multiple comparisons (*p *< 0.05)

^a^Dry basis

### Pretreatment

#### Steam explosion

**Fig. 2:**
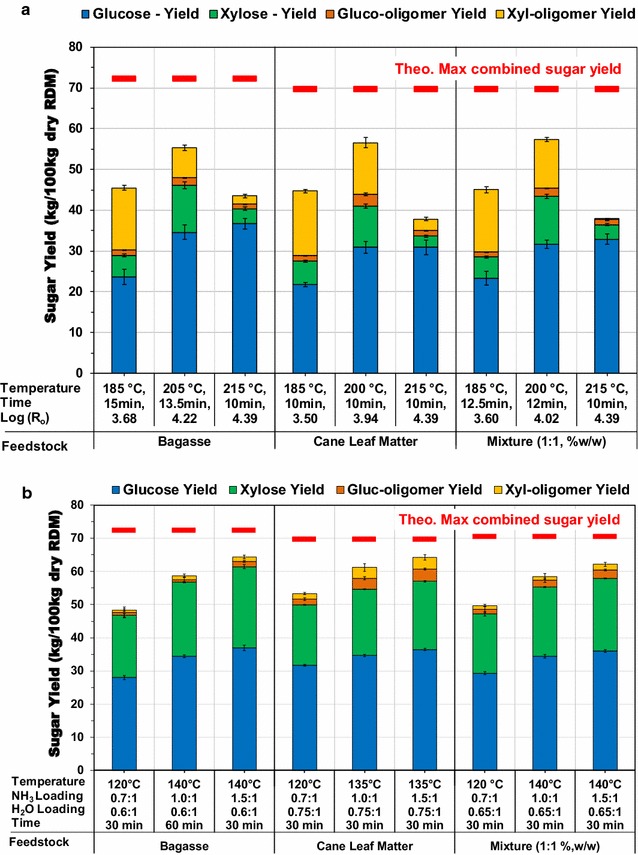
The evaluation of the impact of pretreatment conditions on glucose and xylose yield from sugarcane bagasse, cane leaf matter (CLM) and a bagasse: CLM mixture (1:1 w/w). **a** Steam explosion sugar yield as a function of pretreatment severity. Enzymatic hydrolysis performed at 2% (w/v) WIS loading and incubated 50 °C, for 72 h using 22 mg CTec2 and 11 mg HTec2. **b** AFEX™ sugar yield as a function of temperature, ammonia loading, water loading and residence time. Enzymatic hydrolysis performed at 1% (w/v) glucan loading and incubated 50 °C, for 72 h using 10 mg CTec3 and 5 mg HTec3. Theo.: Theoretical; Max.: Maximum RDM: raw dry material; Log (R_o_): severity factor

The overall glucose and xylose yields from StEx pretreatment at temperatures ranging from 185 to 215 °C and residence times from 10 to 15 min are presented in Fig. 2a. The combined sugar yield was determined from the soluble monomeric and oligomeric sugars (glucose + xylose) in the pretreatment liquor and the soluble monomeric sugars (glucose + xylose) released after enzymatic hydrolysis of washed solids, performed at low solid loading (“Low solids loading enzymatic hydrolysis” section). A summary of the compositions of the pretreated water-insoluble solids, pretreatment liquor and major phenolic compounds in the liquor is presented in Additional file 1: Table S1.

As is common with acid-based pretreatments, increased pretreatment severity successively increased the solubilization of hemicellulose from the plant cell wall matrix, thus enriching the pretreated solids in cellulose and lignin for both bagasse and CLM [30, 33]. Within the evaluated conditions, the highest combined sugar yield for bagasse was obtained at intermediate severity (Log*R*_0_ = 4.22), amounting to 55.3 kg sugar/100 kg RDM (77% of the theoretical maximum). StEx pretreatment of bagasse at this severity facilitated significant hydrolysis of ester linkages in the acetyl group side branches of the xylan backbone as evidenced by an acetic acid yield of 3.36 kg/100 kg RDM in the pretreatment liquor (Additional file 1: Table S1). The accumulation of acetic acid (and other aliphatic and aromatic acids) in the aqueous solution and the presence of hydronium ions from the self-ionization of water at the pretreatment temperature (205 °C, intermediate severity) were reported to catalyse the partial hydrolysis of hemicellulose to soluble hemicellulose monomeric and oligomeric sugars [35, 54]. Accordingly, the total monomeric and oligomeric xylose yield at this condition was 18.9 kg/100 kg RDM (66.1% of the theoretical maximum), with approximately 40% of the xylose recovered in oligomeric form. In comparison, pretreatment at lower severity resulted in a xylose yield of 20.51 kg/100 kg RDM, with more than 74% of the recovered xylose in oligomeric form. However, pretreatment at low severity conditions did not enhance cellulose digestibility as much as the intermediate condition, as demonstrated by a lower glucose yield (57% of the theoretical maximum). Pretreatment at higher severity resulted in the highest glucose yield (86% of the theoretical maximum), but also significant xylan degradation products were produced, likely from the dehydration of xylose and thereby lowered the total sugar yield. Although the intermediate pretreatment severity resulted in the highest combined sugar yield, unavoidable degradation products were nonetheless present in the pentose-rich liquor (Additional file 1: Table S1).

The highest combined sugar yield for the StEx-treated sugarcane CLM was also obtained at the intermediate severity condition (Log*R*_0_ = 3.94), corresponding to 56.5 kg sugar/100 kg RDM (81% of the theoretical maximum). However, unlike StEx-treated bagasse, the highest xylose yield (80.7% of the theoretical maximum) was also obtained at the intermediate severity, with more than 60% of the soluble xylose in oligomeric form. Biomass with high ash content has been previously reported to have some neutralizing/buffering capacity in acidic pretreatments [35, 54]. Untreated CLM is composed of more than 7% ash and about 2% acetyl group content, and therefore, the proton concentration (or [H_3_O^+^]) in the aqueous pretreatment slurry is dependent on competing neutralization, de-acetylation and water ionization reactions. The hydrolysis of insoluble xylan to soluble oligomers is generally observed when the pretreatment temperatures are low or the pH is closer to neutral and the hydrolysis of soluble oligomers to monomeric sugars occurs rapidly under more acidic conditions [33]. As a result, the high ash content and low acetyl group content of CLM may indirectly contribute to the formation of soluble xylan oligomers instead of monomeric xylose, which is prone to dehydration at high temperatures. In support of this hypothesis, we found that the final pH after pretreatment of the CLM at the intermediate severity was 3.7 compared to 3.08 for the bagasse. Consequently, the CLM resulted in higher xylose yield and lower furfural yield relative to the bagasse (Additional file 1: Table S1). Ferrierra-Leita͂o et al. [51] reported a similar finding, with higher buffering capacity and sugar recoveries from CLM relative to sugarcane bagasse for autocatalyzed and CO_2_-impregnated StEx, at pretreatment temperatures similar to those used in this work. Further, pretreating a mixture of bagasse and CLM (at 1:1 ratio on a dry weight basis) at 200 °C and 12 min resulted in a total sugar yield of 57.4 kg sugar/100 kg RDM (82.2% of the theoretical maximum). This outcome suggests that StEx could still be effective for pretreating mixtures of bagasse and CLM when the mean moisture content of the mixture is in the range of 65–75% (w/w).

Based on the combined sugar yield results, the intermediate StEx pretreatment severity for both sugarcane bagasse and CLM were selected as the preferred pretreatment conditions and henceforth used in enzyme cocktail optimization, high solids loading enzymatic hydrolysis and fermentation studies.

#### AFEX™

To understand the interaction of pretreatment parameters and potentially minimize ammonia loading during AFEX™ pretreatment, a wide range of pretreatment conditions were evaluated and statistically modelled. Contour plots and regression models depicting the impact of the pretreatment temperature, ammonia loading and water loading on the combined monomeric sugar (glucose + xylose) yield, following low solids loading enzymatic hydrolysis, are presented in Additional file 3: Fig. S1A, B. The main effects of ammonia loading, pretreatment temperature and water loading were statistically significant and a quadratic, second-order model was sufficient to describe the release of fermentable sugars during enzymatic hydrolysis for AFEX™-treated sugarcane bagasse and CLM, as evident from insignificant lack of fit. The statistically derived regression models were validated by performing additional experiments not included in the original CCD statistical design and subsequently used to predict the combined sugar yield at various ammonia loading conditions, i.e. low, intermediate and high ammonia loadings, as presented in Fig. 2b.

High temperature and high ammonia loading AFEX™ pretreatment resulted in the highest monomeric glucose and xylose yields for both sugarcane bagasse and CLM. The combined monomeric sugar yields achieved at an ammonia loading of 1.5 g NH_3_/g DM were 61.4 kg sugar/100 kg RDM (84.8% of the theoretical maximum) and 57.1 kg sugar/100 kg RDM (81.7% of the theoretical maximum) for sugarcane bagasse and CLM, respectively. High ammonia loading AFEX™ treatment has been shown to enhance the cleavage of ester-linked phenolic compounds in the plant cell wall of monocots, particularly ferulates and coumarates, through ammonolysis reactions [55, 56]. These reactions correlate with higher enzymatic digestion of agricultural grasses [57]. However, high ammonia loadings also translate into higher energy and capital costs for ammonia recovery operations [41, 57]. In comparison, pilot scale AFEX™ pretreatment of corn stover is typically performed at ammonia loadings lower than 1 g NH_3_/g DM [28]. Limiting the ammonia loading to 1 g NH_3_/g DM resulted in combined monomeric sugar yields of 56.8 kg sugar/100 kg RDM (78.3% theoretical maximum) and 54.6 kg sugar/100 kg RDM (78.2% of the theoretical maximum) for sugarcane bagasse and CLM, respectively. While the ammonia loading was reduced by 50%, the combined monomeric sugar yield only reduced by 6.5 and 3.5% for bagasse and CLM, respectively. Although the combined sugar yields for bagasse and CLM were quite similar, the CLM glucan conversion (83% of the theoretical maximum) was less sensitive to the reduced ammonia loading relative to the bagasse (77% of the theoretical maximum). Oligomeric analysis of the CLM enzymatic hydrolysate revealed significant quantities of xylooligomers, hinting at the possible absence of some auxiliary activities in the enzyme cocktail employed, which may be required to further increase the combined sugar yields for CLM from AFEX™ pretreatment [58].

The statistically derived regression models were used to identify pretreatment conditions that would be suitable for the AFEX™ pretreatment of a mixture of bagasse and CLM (Additional file 4: Fig. S2). AFEX™ pretreatment of a bagasse-CLM mixture (composed of 1:1 w/w ratio) at 140 °C, 1 g NH_3_/g DM, 0.65 g H_2_O/g DM and 30 min residence time produced a combined monomeric sugar yield of 55.3 kg sugar/100 kg RDM (78.3% of the theoretical maximum). Like StEx pretreatment, this result demonstrates the suitability of AFEX™ to sugarcane residue mixtures, provided the initial moisture of the mixture prior to ammonia addition is approximately 0.65 g H_2_O/g DM.

Packed-bed AFEX™ pretreatment on pilot scale is designed to receive biomass with an initial moisture content of approximately 30% before being pre-steamed to simultaneously preheat the biomass and adjust the moisture content to an optimized water loading (typically 60–70%) [28]. In industry, sugarcane bagasse fed into mill boilers is obtained after juice extraction, warm washing and dewatering operations and usually has a moisture content of approximately 50–60%. Surplus bagasse is typically stockpiled for storage and occasionally mildly irrigated to minimize the risk of spontaneous combustion [59]. Therefore, for current AFEX™ pretreatment designs, energy would need to be expended to dry the bagasse towards a lower moisture content prior to pretreatment. Previously, it was shown that AFEX™ pretreatment of high-moisture content bagasse required an ammonia to biomass loading of 2.0 g NH_3_/g DM to achieve glucan conversions greater than 75%, demonstrating the necessity of lowering the moisture content of bagasse prior to pretreatment [60]. In contrast, CLM is likely to be left on the field and allowed to dry down to moisture levels where it can be easily managed. In general, dried CLM typically has a much lower moisture content (about 15%) and therefore would be at a much more suitable moisture content for direct use in AFEX™ pretreatment. Alternatively, mixing these two substrates may negate the need for expending significant energy for drying the bagasse and/or minimize water consumption for adjusting the initial moisture of the CLM prior to AFEX™ pretreatment. As suggested by the results in this section, mixing these two substrates at appropriate ratios would not significantly affect the pretreatment effectiveness as measured by the combined monomeric sugar yields from downstream enzymatic hydrolysis, thus making AFEX™ also agnostic to sugarcane residues. Ultimately, local biomass harvesting techniques (manual vs mechanical), logistics, handling and storage infrastructure available at the biorefinery will likely define processing decisions (e.g. on-field CLM drying, milling operations) necessary to minimize energy expenditure for maximizing AFEX™ or StEx pretreatment efficiency.

### High solids loading enzymatic hydrolysis

Due to uncertainties regarding the cost of enzymes, minimizing the enzyme dosage would ensure that AFEX™- or StEx-based biorefineries would be less sensitive to fluctuations in enzyme purchase or on-site production costs [61]. High solids loading enzymatic hydrolysis (HSL-EH) was evaluated to compare the effect of enzyme dosage and solids processing option on monomeric sugar yields based on configurations defined in Fig. 1. Optimal commercial enzyme combinations (CTec3: HTec3: Pectinex Ultra-SP) for AFEX™- and StEx-treated bagasse and CLM were used to maximize the saccharification yields for each pretreated substrate (Additional file 5: Fig. S3). The corresponding glucose and xylose yields were based on the weight of monomeric sugars recovered relative to the total weight of the corresponding carbohydrate loaded.

**Fig. 3:**
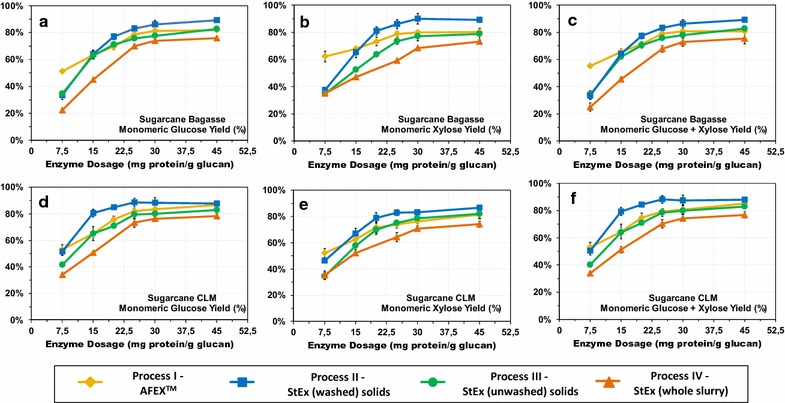
Glucose and xylose yields for high solids loading enzymatic hydrolysis of AFEX™, StEx (washed solids), StEx (unwashed solids), and StEx (whole slurry) treated sugarcane bagasse and CLM. Enzymatic hydrolysis was performed using optimized mixtures of CTec3, HTec3 and Pectinex Ultra-SP. The solids loading was maintained at 10% carbohydrate loading, pH 4.8 and incubated at 50 °C for 96 h. The glucose yields were calculated based on the weight of monomeric glucose recovered relative to the sum of the weight of the insoluble glucan and soluble G-OS content at the beginning of the enzymatic hydrolysis. Similarly, the xylose yields were calculated based on the weight of monomeric xylose recovered relative to the insoluble xylan and soluble X-OS. The combined glucose + xylose yields were calculated based on the sum of the monomeric glucose and xylose recovered relative to the sum of insoluble glucan, insoluble xylan, soluble G-OS and soluble X-OS

The monomeric glucose and xylose yields as a function of the enzyme dosage are presented in Fig. 3. AFEX™-treated bagasse and CLM (Process I) achieved glucose yields of 77% and 81.5% at the inflection enzyme dosage of 25 mg g^−1^ glucan, respectively. However, an additional 16 and 14% of the total sugars released from AFEX™-treated bagasse and CLM were in oligosaccharide form, respectively (data not shown). At lower enzyme loadings, the accumulation of these soluble oligosaccharides was even more pronounced. For example, the monomeric glucose and xylose yields for AFEX™-treated CLM at 15 mg enzyme g^−1^ glucan were 65 and 63%, respectively. However, an additional 14% G-OS and 21% X-OS were recovered in the enzymatic hydrolysate. The accumulation of oligomeric sugars is not unique to AFEX™ pretreatment and has also been demonstrated for dilute acid and ionic liquid-pretreated corn stover [58]. These soluble oligomeric sugars not only inhibit the activity of commercial enzyme mixtures, but they also represent lost yield since most ethanologens only consume monomeric sugars [62]. The discovery of enzyme activities that are absent from current commercial cocktail mixtures for converting recalcitrant oligosaccharides to fermentable monomeric sugars can potentially generate higher fermentable sugar yields or even reduce enzyme requirements for these AFEX™-treated sugarcane residues [63].

For StEx pretreatment, the presence of organic acids, furan aldehydes, phenolic compounds and soluble sugars (monomeric and oligomeric) limited the enzyme activity and subsequently required high enzyme dosages to achieve high sugar yields (Additional file 1: Table S1). This was evident as separating and washing StEx-pretreated bagasse or CLM solids (Process II) resulted in higher combined glucose plus xylose yields relative to unwashed solids or the whole slurry processing options (Process III and IV). Washing StEx solids has been reported to remove some of the inhibitory components including soluble carbohydrates (especially X-OS and monomeric sugars), soluble organic acids, water-soluble aromatics and furan derivatives that might have adsorbed onto the solid biomass during pretreatment [46, 64, 65]. Interestingly, for CLM, washing the solids had a larger impact on the glucose and xylose yields at lower enzyme loadings. At 15 mg g^−1^ glucan, the combined glucose plus xylose yield for StEx-CLM (washed) solids was 80% relative to 64 and 51% for unwashed solids and whole slurry, respectively (Fig. 3f). StEx-treated CLM produced a pretreatment liquor that was rich in oligosaccharides (particularly X-OS) that strongly inhibit cellulases (particularly CBH I and CBH II) [58, 66]. Hence, by introducing a solid–liquid separation step and/or washing the StEx-pretreated CLM solids, the effect of enzyme inhibition by soluble X-OS or degradation products can be minimized, and enzyme loadings can be significantly reduced. At lower enzyme loadings (< 15 mg g^−1^ glucan), the glucose and xylose yields from StEx-treated bagasse and CLM both with washing and without washing decreased sharply. This effect could be due to end-product inhibition, enzyme access blockage by lignin and/or non-productive binding of the hydrolytic enzymes to lignin [67].

Given that the enzyme costs were previously estimated to account for 15.7% of the total costs even at enzyme loadings of 20 mg g^−1^ glucan, it may be necessary to explore processing options that further reduce the required enzyme dosage [68]. As demonstrated in this work, depending on the pretreatment conditions and the pretreated biomass, investing in solid–liquid separation and/or washing steps may reduce the enzyme loadings. However, an economic and environmental impact assessment may be necessary to decide whether the enzyme savings for using washed solids outweigh the requirements for additional capital and operating costs for solid–liquid separation and/or washing operations. Similarly, lowering the enzyme loading for AFEX™-treated bagasse or CLM may also require an economic and environmental impact assessment given that by altering the pretreatment conditions (e.g. using a higher ammonia loading during pretreatment), the enzyme loading requirements to reach target sugar yields can be lowered at the expense of higher capital and operational costs for ammonia recovery.

### Fermentation

**Fig. 4:**
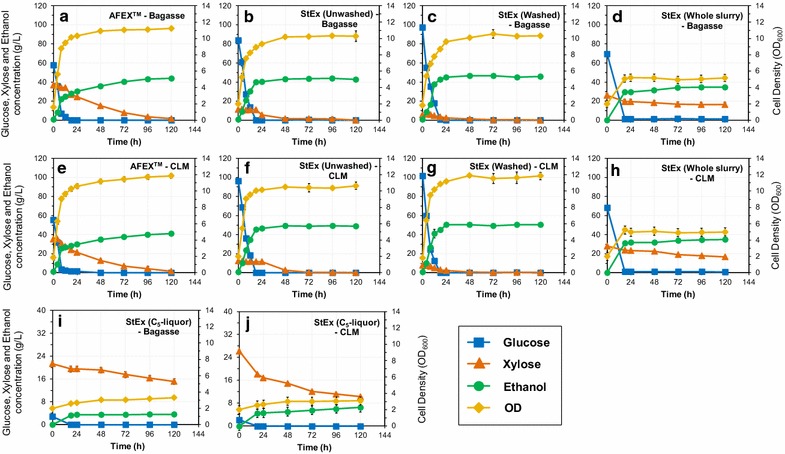
Fermentation time profiles for enzymatic hydrolysates obtained from AFEX™, StEx (washed solids), StEx (unwashed solids), StEx (whole slurry) and StEx (C_5_-liquor) sugarcane bagasse and cane leaf matter. Fermentations were performed using *S. cerevisiae* 424A (LHN-ST) with an initial inoculum of 0.96 g CDW L^**−**1^ at 30 °C, pH 5.5, and a shaking speed of 150 rpm for 120 h. All enzymatic hydrolysates were supplemented with 0.25% (w/w) corn steep liquor prior to fermentation. Square—glucose (g L^**−**1^), Triangle—xylose (g L^**−**1^), Circle—ethanol (g L^**−**1^), Diamond—OD_600nm_

The fermentation profiles for converting enzymatic hydrolysates and the StEx C_5_-liquor (configurations shown in Fig. 1) to ethanol are presented in Fig. 4. A summary of the fermentation performance of xylose-fermenting *S. cerevisiae* 424A (LNH-ST) on the various process streams is presented in Table 2. The extent of glucose or xylose consumption, ethanol metabolic yield and ethanol titre were used as metrics for comparing the fermentability of the various streams.

**Table 2:** Summary of fermentation parameters of steam-exploded and AFEX™-treated bagasse and cane leaf matter

Parameter	AFEX™—bagasse	StEx—bagasse—unwashed solids	StEx—bagasse—washed solids	StEx—bagasse—whole slurry	StEx—bagasse—C_5_-liquor	AFEX™—CLM	StEx—CLM—unwashed solids	StEx—CLM—washed solids	StEx—CLM—whole slurry	StEx—CLM—C_5_-liquor
Initial glucose conc. (g L^−1^)	59.03	83.89	97.56	69.67	2.89	58.45	96.27	101.49	68.30	2.10
Initial xylose conc. (g L^−1^)	37.01	11.22	5.60	26.09	21.19	35.15	12.38	7.70	27.95	26.18
Glucose consumption (%)	100	100	100	98	100	100	100	100	99	100
Xylose consumption (%)	96	90	95	37	29	95	97	96	41	61
µ_max_ (h^−1^)^a^	0.31	0.24	0.27	0.05	0.013	0.3	0.25	0.32	0.055	0.015
Y _x/s_ (g CDW/g sugar)^b^	0.05	0.04	0.04	0.03	0.02	0.05	0.04	0.04	0.03	0.01
Metabolic yield (%)^c^	92	91	89	87	69	89	89	90	87	71
Y_p/s_ (g EtOH/g sugar)^d^	0.46	0.45	0.45	0.36	0.13	0.44	0.45	0.46	0.36	0.23
EtOH conc. (g L^−1^)	44.17	43.48	46.75	34.62	3.18	41.70	49.24	50.21	35.07	6.48
EtOH yield (kg/100 kg DM)^e^	25.60	16.54	16.84	16.23	1.69	24.90	15.98	16.58	16.67	3.75

All hydrolysates were supplemented with 0.25% (w/w) corn steep liquor prior to fermentation. The C_5_-liquor/s were acid hydrolysed to convert oligosaccharides to their monomeric counterparts prior to fermentation

Data represent the averages of independent duplicate fermentation cultivations. All standard errors were less than 5%

^a^Maximum specific growth rate: maximum growth rate calculated in the exponential growth phase

^b^Cell biomass yield: gram of cell dry weight per gram of sugar (glucose + xylose) consumed during fermentation

^c^Metabolic yield: gram of ethanol produced per gram of sugar (glucose + xylose) consumed during fermentation

^d^Ethanol yield: gram ethanol produced per gram of sugar at the beginning of fermentation

^e^Ethanol yield per 100 kg of untreated dry material

Like most native *S. cerevisiae* strains, the microbial strain used in this work typically demonstrates slow diauxic xylose fermentation due to the lack of high-affinity xylose transporters in the presence of glucose [69]. As a result, glucose was rapidly consumed from all process streams (Process I–IV) within 18 h (Fig. 4). In agreement with previous reports, AFEX™-derived bagasse and CLM enzymatic hydrolysates (Process I) achieved near complete xylose consumption, with ethanol metabolic yields and ethanol titres greater than 89% and 40 g L^−1^, respectively [70, 71]. Similarly, near complete xylose consumption was observed for washed and unwashed StEx-derived bagasse and CLM enzymatic hydrolysates (Process II and III), with approximately 90% metabolic yield and ethanol titres greater than 40 g L^−1^. Moreover, the fermentation of these enzymatic hydrolysates was complete after 48 h owing to their low initial xylose concentrations (< 15 g L^−1^) and the supplementation with corn steep liquor. This observation is supported by previous work that demonstrated that xylose fermentation performance of *S. cerevisiae* 424A (LNH-ST) was influenced by nutrient availability in the fermentation media [40]. The fermentation of whole slurry enzymatic hydrolysates (Process IV) resulted in significantly lower xylose consumption and slightly lower metabolic yield for both StEx-treated bagasse and CLM. Whole slurries derived from acidic pretreatments are typically rich in various pretreatment inhibition products, including aliphatic and aromatic carboxylic acids, furan aldehydes and phenolic compounds [46]. Moreover, because glucose fermentation occurs before xylose fermentation, ethanol and other accumulated fermentation metabolites generated during the glucose consumption phase further inhibit xylose fermentation. The presence of fermentation metabolites has been previously shown to play a critical role in inhibiting xylose uptake by *S. cerevisiae* 424A (LNH-ST) [40, 72]. Therefore, the limited xylose fermentation performance for StEx-whole slurries may be attributed to the inability of this strain to buffer redox changes caused by the synergistic/combined effect of pretreatment inhibitors, ethanol and fermentation metabolites [73, 74]. Nonetheless, even in the presence of microbial inhibition, ethanol titres of approximately 35 g L^−1^ at metabolic yields greater than 85% were achieved for both StEx bagasse and CLM whole slurries. In comparison, Mosier et al, [75] reported metabolic yields and a final ethanol concentration of 88% and 22.5 g L^−1^, respectively, for the fermentation of non-detoxified liquid hot water-treated corn stover whole slurry hydrolysate by *S. cerevisiae* 424A (LNH-ST).

The StEx bagasse and CLM C_5_-liquor streams were poorly fermented by *S. cerevisiae* 424A (LNH-ST), as demonstrated by low specific growth rate, xylose consumption and ethanol yield compared to the enzymatic hydrolysates. Like the StEx-whole slurries, it appears that this yeast strain’s fermentation performance was limited by degradation product inhibition. Although C_5_-liquor fermentation was limited due to microbial inhibition, xylose consumption was not completely arrested as xylose was still being consumed albeit at a significantly slower rate (approx. 0.05 g L^−1^ h^−1^) after 120 h (see Fig. 4i, j). Recently, recombinant *S. cerevisiae* strains MEC1122 and LF1 demonstrated ethanol yields up to 0.42 g g^−1^ in non-detoxified liquid hot water-treated corn cob C_5_-liquor and StEx-treated corn stover hydrolysate, respectively [76, 77]. Therefore, developing hardened xylose-fermenting mutant strains with higher tolerance of pretreatment-derived inhibitors and fermentation metabolites could hypothetically improve fermentation yields and ethanol titres from the C_5_-liquor streams [78].

The recovery of X-OS via a dilute acid post-hydrolysis of the C_5_-rich liquid fraction is an example of a process that can be performed in a simple stirred-tank or plug flow reactor in a commercial setting, without the need for a complex high solids reactor configuration [79]. This option is particularly important because smaller reaction volumes will be necessary since only the pseudo-homogenous liquid fraction will be hydrolyzed. Moreover, the post-hydrolysis is performed at much lower reaction temperatures (∼ 120 °C) without the threat of significant ash neutralization by high ash content biomass slurries. Hence, capital expenses can be reduced due to requirement of a significantly smaller reactor that is lined with resistant but high-cost anti-corrosion alloys. Another pertinent issue with the StEx C_5_-liquor stream is the dilute concentration of total sugars available for fermentation. The sugar concentration of this stream can potentially be increased by increasing the solids loading during StEx pretreatment. However, increasing the solids loading is usually coupled with lower pretreatment efficiency and higher concentrations of organic acids, particularly acetic acid, which is a major microbial inhibitory compound. On the other hand, the C_5_-liquor stream could be concentrated using thermal evaporation technology, similar to that applied for concentrating cane juice, to remove water and volatile products such as acetic acid and furan derivatives. However, such an approach would likely increase operating costs and enrich the C_5_-liquor stream with other non-volatile inhibitory compounds such as vanillin and coniferyl aldehyde [46].

For 2G biorefineries annexed to 1G autonomous distilleries or existing sugar mills, the C_5_-liquor stream could be mixed with molasses or sugarcane juice to simultaneously increase the total stream sugar concentration and dilute the concentration of the pretreatment-derived inhibitors. Losordo et al. [79] reported that up to 37% more ethanol could be produced without affecting sugar coproduction when the C_5_-sugars from StEx are combined with molasses. Therefore, with adequate process integration and yeast development, there are potential avenues to convert these C_5_-sugars into ethanol or other commodity chemicals.

### Process mass balances

**Fig. 5:**
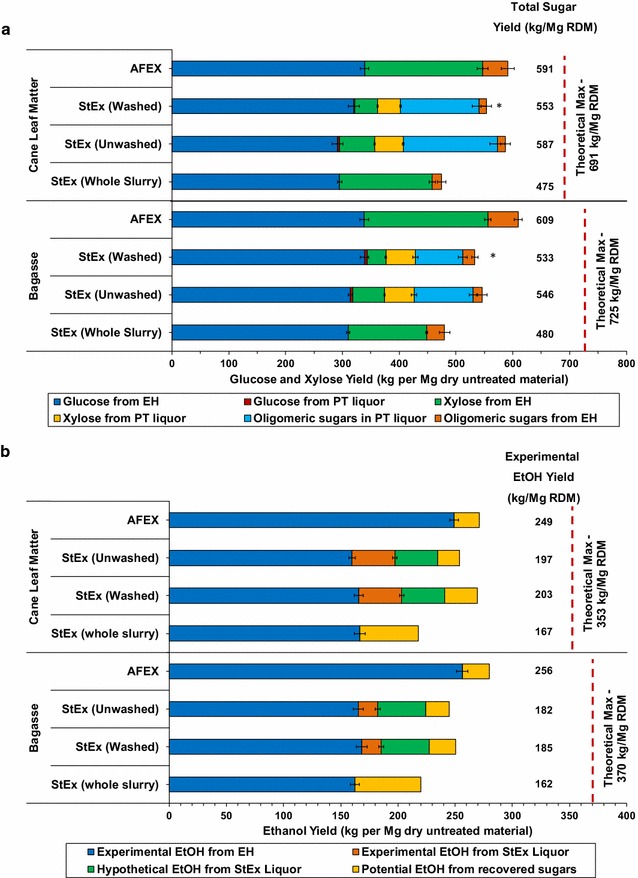
Comparison of bagasse and CLM carbohydrate recovery (**a**) and ethanol yields (**b**) per ton dry biomass from AFEX™ and StEx pretreatment coupled with various solids processing options. The theoretical maximum carbohydrate recovery and ethanol yields (red dotted line) were calculated based on the initial glucan and xylan content in untreated bagasse and CLM). The solids loading was maintained at 10% carbohydrate loading, pH 4.8, and enzyme dosage at 25 mg g^**−**1^ glucan and incubated at 50 °C for 96 h. *Assumed that sugars removed from washing step are directly sent to wastewater treatment and therefore not considered as recovered sugars

The results from pretreatment, high solids loading enzymatic hydrolysis at 25 mg g^−1^ glucan and fermentation were used to develop mass balances for each biomass material in each process configuration (Process I–IV). The carbohydrate recovery of monomeric and oligomeric sugars from pretreatment and HSL-EH relative to the initial untreated dry material is presented in Fig. 5a, whereas the ethanol yield from the recovered carbohydrates is presented in Fig. 5b. Detailed process flow diagrams are presented in Additional file 6: Fig. S4.

For bagasse, AFEX™ pretreatment ultimately generated the highest carbohydrate recovery (609 kg sugar/ton RDM or 84% theoretical maximum), owing to absence of significant polysaccharide degradation during pretreatment and high enzymatic hydrolysis conversion of both glucan and xylan. AFEX™ consumed about 15 kg of ammonia Mg^−1^ RDM, primarily due to ammonolysis reactions with the biomass and residual ammonia chemically bound to the biomass, which would have to be replenished after every cycle on an industrial scale. The remaining ammonia can be recycled and reused as demonstrated at MBI International’s pilot plant operation [80]. About 7% of the recovered carbohydrates were in oligomeric form, which highlights the importance of identifying enzyme activities absent from current commercial enzyme mixtures required to maximize ethanol production from these residues. For StEx-pretreated bagasse, unwashed solids generated the highest carbohydrate recovery (546 kg sugar/Mg RDM or 75% theoretical maximum). Although washed solids achieved the highest enzymatic hydrolysis conversions, washing the solids removed about 21 kg water-soluble monomeric sugars and oligosaccharides per Mg RDM. Moreover, washing the StEx solids with water heated to 50 °C consumed approximately 10 kg of water kg^−1^ of unwashed solids, thereby increasing the overall process water consumption. Although we considered the water-soluble sugars as “lost” sugars in carbohydrate recovery calculations, in a biorefinery setting, it is likely that these sugars would be sent directly to an anaerobic digestion-based wastewater treatment to produce process energy in the form of methane. Further, on an industrial scale, part of the wash water would be recycled to wash next batch of StEx-treated solids and thus reduce the overall water requirements for the washing step. The StEx-whole slurry produced the lowest carbohydrate recovery (480 kg sugar Mg^−1^ RDM or 66% theoretical maximum) due to significant enzyme inhibition during HSL-EH. Moreover, approximately 4% of the solubilized sugars were retained in oligomeric form (the highest among the StEx solids processing options).

For CLM, AFEX™ and StEx-unwashed solids resulted in the highest carbohydrate recoveries of 591 kg sugar Mg^−1^ RDM and 587 kg sugar Mg^−1^ RDM, respectively. The difference between the two process configurations, Process I and III, was statistically insignificant (*p* > 0.05). Similar to the case of StEx-treated bagasse, washing StEx-treated CLM removed approximately 37 kg of soluble sugars Mg^−1^ RDM and therefore resulted in the recovery of 553 kg sugar Mg^−1^ RDM. However, these mass balances were performed at relatively higher enzyme loadings (25 mg g^−1^ glucan), which imply that the benefit of washing the StEx solids on the carbohydrate recovery may become more apparent at lower enzyme loadings (e.g. at 15 mg g^−1^ glucan). Finally, StEx-CLM whole slurries recovered the least glucan and xylan due to a combination of sugar degradation during pretreatment and enzyme inhibition during HSL-EH.

The ethanol yield per ton of RDM provides a means of quantifying the combined effect of biomass recalcitrance, enzyme inhibition and microbial inhibition for the various AFEX™ and StEx process configurations. AFEX™-treated bagasse and CLM (Process I) generated the highest ethanol yields due to higher sugar recovery and superior fermentability of the AFEX™ hydrolysates by *S. cerevisiae* 424A (LNH-ST) without detoxification. The estimated ethanol yields for AFEX™ bagasse and CLM were 256 and 249 kg ethanol Mg^−1^ RDM, respectively. The poor fermentability of the StEx C_5_-liquor and whole slurry significantly impacted the ethanol yield for StEx bagasse, resulting in 185, 182 and 162 kg ethanol Mg^−1^ RDM recovered from Processes II, III and IV, respectively. The experimental ethanol yields for StEx-CLM were 203, 197 and 167 kg ethanol Mg^−1^ RDM for Process II, III and IV, respectively. These ethanol yields were slightly higher than those of bagasse due to higher ethanol yield from the C_5_-liquor derived from CLM relative to that derived from bagasse. In general, the StEx bagasse C_5_-liquor contained higher concentrations of well-known microbial inhibitors, including organic acids and phenolic compounds, thus producing lower ethanol yields relative to the StEx-CLM-generated liquor (Additional file 1: Table S1). Nonetheless, the lower StEx ethanol yields relative to AFEX™ demonstrate the compounded consequences of sugar loss due to degradation during StEx pretreatment, the degree of enzyme inhibition due to the solids processing option and microbial inhibition of *S. cerevisiae* 424A (LNH-ST) due to the presence of pretreatment-derived inhibitors.

### Estimation of 2G ethanol yields per sugarcane cultivation area

In this work, we developed comprehensive process mass balances, based on experimental data, to estimate the potential ethanol yields that can be achieved at industrially relevant conditions from 2G sugarcane-based biorefineries using mature technologies available today (Additional file 6: Fig. S4). Assuming a commercial average sugarcane yield of 80 metric ton of wet cane per hectare, it was estimated that AFEX™-based biorefineries (Process I) would generate higher ethanol yields per sugarcane cultivation area (4496 L ha^−1^) relative to StEx-based biorefineries (3416–3341 L ha^−1^), irrespective of the StEx processing configuration (Table 3) [81]. As previously discussed, StEx process bottlenecks that lowered the ethanol yields were mainly associated with sugar degradation during pretreatment, enzyme inhibition and the inability of recombinant *S. cerevisiae* 424A (LNH-ST) to efficiently convert the sugars in the C_5_-liquor to ethanol.

**Table 3:** Estimated ethanol yield per hectare of sugarcane cultivation area

	Yield
Sugarcane crop segment	
Average cane yield (Mg wet cane ha^−1^)	80.0
Bagasse (kg dry fibre Mg^−1^ wet cane)^a^	140.0
Available bagasse (Mg dry fibre ha^−1^)^b^	11.2
Cane leaf matter (kg dry fibre Mg^−1^ wet cane)^a^	140.0
Available cane leaf matter (Mg dry fibre/Mg wet cane)^c^	5.6
2G Bagasse + CLM—ethanol yield (L ha^−1^)^d^	
AFEX™—Process I	4496
StEx (washed solids + C_5_-liquor)—Process II	3416
StEx (unwashed solids + C_5_-liquor)—Process III	3341
StEx (whole slurry)—Process IV	2911

^a^Estimated sugarcane bagasse and cane leaf matter yield per ton wet cane [16]

^b^Assuming 75% of bagasse collected from the sugar mill is allocated to biofuel production and the remainder will supplement lignin-rich enzymatic residues for energy cogeneration

^c^Assuming 50% of the CLM harvested on the field can be removed without significantly affecting soil fertility [18–21]

^d^Ethanol yield calculated by multiplying available bagasse or CLM (Mg dry fibre ha^−1^) with the experimental ethanol yield (L Mg dry fibre^−1^)

**Fig. 6:**
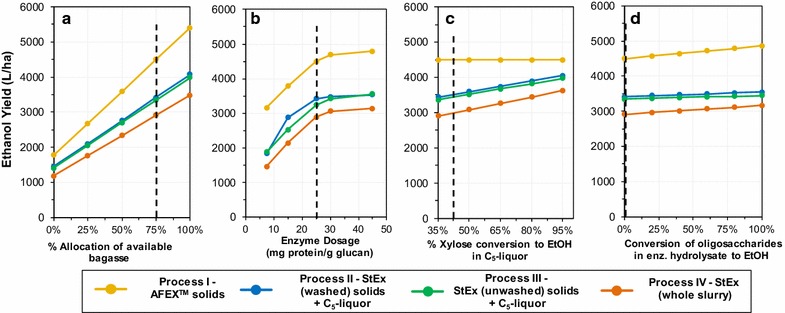
Sensitivity analysis on the ethanol yield per hectare of sugarcane cultivation area considering: **a** percent allocation of available bagasse, **b** the enzyme dosage, **c** percent of xylose conversion to ethanol from the C_5_-liquor, **d** the conversion of the oligosaccharides in the enzymatic hydrolysate to ethanol. Black dotted line indicates baseline conditions which were selected based on experimental data presented in Table 2 and the assumptions presented in Table 3

A single variable sensitivity analysis was carried out to project the effect of sugarcane bagasse allocation, variation in enzyme dosage, the extent of xylose conversion to ethanol in the StEx C_5_-liquor and the conversion of oligosaccharides present in the enzymatic hydrolysate on the estimated ethanol yields from AFEX™ or StEx per sugarcane cultivation area (Fig. 6). The quantity of bagasse allocated to biofuel production had the highest impact on the ethanol yield, with AFEX™ yields decreasing from 4496 to 3586 L ha^−1^ when the quantity of available bagasse allocated to ethanol production is reduced from 75 to 50% (Fig. 6a). End-of-process sugarcane bagasse from the sugar mill will be required to supplement lignin-rich enzymatic hydrolysis residues to provide process energy for the 2G biorefinery, hence the amount of bagasse available for biofuel production will be limited by factors such as the state of mill boiler technology and the biorefinery plant size [82]. A recent study estimated that bagasse allocation for ethanol production in Brazil ranges from 64 to 84% depending on the electricity production cost, ethanol production cost, plant size and regulation of the electricity/biofuel markets [83]. A similar study revealed that 65% of the available sugarcane residues (bagasse and CLM) could be allocated to biofuel production, with the remainder being diverted towards energy cogeneration operations to ensure that South African sugar mills meet their steam and energy demands [82].

Reducing the enzyme dosage below 25 mg g^−1^ glucan lowered the ethanol yields for all processes (Process I to IV), with Process IV (whole slurry) obtaining the lowest yield of 1458 L ha^−1^ at an enzyme dosage of 7.5 mg protein g^−1^ glucan (Fig. 6b). In comparison, AFEX™ (Process I) was estimated to achieve an ethanol yield of 3154 L ha^−1^ at the same enzyme dosage (more than double that of Process IV). This result demonstrates again the compounded effect of enzyme and microbial inhibition during whole slurry processing. Increasing xylose to ethanol conversion from the C_5_-liquor improved ethanol yields for Processes II to IV from 2849 to 4045 L ha^−1^ when hypothetical xylose consumption and metabolic yield scenarios of 95 and 90% were considered, respectively. Therefore, by using a suitable hardened xylose-fermenting yeast or even exploring process integration strategies such as mixing the C_5_-liquor stream with sugarcane molasses, ethanol yields can be significantly improved for StEx-treated sugarcane residues to approach those achieved by AFEX™-treated residues. Lastly, the conversion of all recalcitrant oligosaccharides from enzymatic hydrolysates to ethanol would significantly improve ethanol yields from AFEX™-treated residues from 4496 to 4860 L ha^−1^. In contrast, minor increments in the ethanol yield would be achieved with increasing oligosaccharide conversion to ethanol in StEx hydrolysates. Hence, identifying auxiliary enzymatic activities missing from the commercial cocktails used in this work could benefit AFEX™-treated residues more than StEx-treated residues.

## Conclusions

In the context of expanding the sugar industry towards a diversified bioeconomy, the use of sugarcane harvest residues (including bagasse and cane leaf matter) in a 2G biorefinery presents an attractive opportunity for increasing ethanol yields per unit of land cultivated, while facilitating the sharing of existing logistics and supply chain infrastructure with the sugar industry. In this work, we evaluated the ethanol production potential for future sugarcane residue-based biorefineries with AFEX™ or StEx as the central pretreatment technologies. AFEX™ proved to be the more effective pretreatment technology for maximizing ethanol yields from sugarcane residues, resulting in ethanol yields of 249 and 256 kg Mg^−1^ RDM (equivalent of 316–325 L Mg^−1^ RDM) for sugarcane bagasse and CLM, respectively. In comparison, steam explosion-pretreated sugarcane bagasse and CLM generated 162–203 kg of ethanol Mg^−1^ RDM (205–257 L Mg^−1^ RDM) depending on the solids processing option chosen to follow pretreatment.

Although both pretreatments were agnostic for sugarcane residues, we identified some process limitations for both technologies. Currently, both pretreatments required relatively high enzyme loadings (> 20 mg g^−1^ glucan) to reach carbohydrate conversions greater than 75%, even with some of the most efficient commercial enzyme combinations. Due to uncertainties in the enzyme cost, the enzyme usage for both pretreatments would need to be reduced to decrease the sensitivity of these biorefineries to enzyme cost fluctuations. Moreover, ethanol yields from StEx-treated bagasse and CLM were limited by a combination of sugar degradation during pretreatment, enzyme inhibition and the inhibition of recombinant *S. cerevisiae* 424A (LNH-ST) due to pretreatment-derived inhibitors. On the other hand, hydrolysis of AFEX™-treated bagasse and CLM left more than 7% of the total sugars in oligomeric form, thereby reducing the overall sugar and ethanol yields.

Overall, selecting the preferred pretreatment technology is primarily an economic and environmental impact issue. Hence, estimating the cost of ethanol production ($USD/L ethanol) through techno-economic analysis and environmental impacts through a life-cycle analysis would provide the necessary basis for comparing 2G sugarcane biorefineries centred on AFEX™ or StEx pretreatment. This work provides insights that will enable later economic and environmental evaluations of the impacts of the various AFEX™/StEx processing options on the cost of ethanol production.

## Additional files

**Additional file 1: Table S1.** Composition of the liquid and solid fractions after water-impregnated steam explosion of sugarcane bagasse, cane leaf matter and a bagasse-CLM mixture (at 1:1 w/w ratio).

**Additional file 2: Table S2.** AFEX™ -bagasse pretreatment conditions used for evaluating the effect of pretreatment conditions on the monomeric glucose, xylose and combined sugar yield using a central composite design of experiments (DOE).

**Additional file 3: Fig. S1.** Design of experiments results for evaluating the effect of AFEX™ pretreatment conditions on the monomeric combined sugar yield from sugarcane bagasse and CLM.

**Additional file 4: Fig. S2.** Profiling the effect of ammonia loading and temperature on the combined glucose and xylose yields for AFEX™-treated bagasse and cane leaf matter after 1% glucan loading enzymatic hydrolysis with 15 mg protein g^−1^ glucan.

**Additional file 5: Fig. S3.** Statistical optimization of Cellic^®^ CTec3, Cellic^®^ HTec3, Pectinex Ultra-SP combinations for maximizing combined glucose and xylose yields from AFEX^TM^ and Steam exploded sugarcane bagasse and CLM.

**Additional file 6: Fig. S4.** Material balances during pretreatment, washing, hydrolysis and fermentation for Processes I–IV.

## Abbreviations

AFEX™: ammonia fibre expansion
ANOVA: analysis of variance
CLM: cane leaf matter
G-OS: gluco-oligosaccharides
HHV: higher heating value
HPLC: high-performance liquid chromatography
HSL-EH: high solids loading enzymatic hydrolysis
LAP: laboratory analytical procedure
LCC: lignin-carbohydrate complex
MBI: Michigan Biotechnology Institute
OD: optical density
SHF: separate hydrolysis and fermentation
StEx: steam explosion
WIS: water-insoluble solids
WSS: water-soluble solids
X-OS: xylo-oligosaccharides
YEP: yeast extract peptone
