# Supplementary material for: Ethanol production potential from AFEX™ and steam-exploded sugarcane residues for sugarcane biorefineries
Source: Biotechnol Biofuels. 2018 May 4;11:127. doi: 10.1186/s13068-018-1130-z (PMC5934847; doi:10.1186/s13068-018-1130-z)
Supplement: Supplementary file 1 — Additional file 1: Table S1. Composition of the liquid and solid fractions after water-impregnated steam explosion of sugarcane bagasse, cane leaf matter and a bagasse-CLM mixture (at 1:1 w/w ratio). [file 13068_2018_1130_MOESM1_ESM.docx]

**Additional File 1**

Table S1: Composition of the liquid and solid fractions after water-impregnated steam explosion of sugarcane bagasse, cane leaf matter and a bagasse-CLM mixture (at 1:1 w/w ratio). Each pretreatment condition was performed in duplicate or triplicate. Mean and standard deviation of triplicate experiments are reported.

| **Pretreatment Conditions** | **Bagasse** | | | | **CLM** | | | | **Bagasse + CLM Mixture** | | | |
| --- | --- | --- | --- | --- | --- | --- | --- | --- | --- | --- | --- | --- |
|  | **Raw** | **LS - StEx** | **MS-StEx** | **HS - StEx** | **Raw** | **LS - StEx** | **MS-StEx** | **HS - StEx** | **Raw** | **LS - StEx** | **MS-StEx** | **HS - StEx** |
| Temperature (°C) | - | 185.0 | 205.0 | 215.0 | - | 185.0 | 200.0 | 215 | - | 185.0 | 200.0 | 215.0 |
| Residence Time (min) | - | 15.0 | 13.5 | 10.0 | - | 10.0 | 10.0 | 10.0 | - | 12.5 | 12.0 | 10.0.0 |
| Severity Factor | - | 3.68 | 4.22 | 4.39 | - | 3.50 | 3.94 | 4.39 | - | 3.60 | 4.02 | 4.39 |
| **Solids Calorific Value (MJ/kg)** | | | | | | | | | | | | |
| HHV | 18.5 ± 0.06 | 19.2 ± 0.08 | 19.9 ± 0.10 | 20.2 ± 0.09 | 17.7 ± 0.10 | 18.5 ± 0.03 | 18.9 ± 0.06 | 19.4 ± 0.00 | 17.9 ± 0.10 | 18.9 ± 0.0 | 19.4 ± 0.15 | 19.9 ± 0.00 |
| **Component in Solids (g/100g RDM)** | | | | | | | | | | | | |
| WIS Recovery | 100.0 | 68.9 ± 1.92 | 63.1 ± 1.6 | 59.3 ± 1.22 | 100.0 | 71.6 ± 1.02 | 59.9 ± 1.58 | 58.1 ± 1.00 | 100.0 | 67.7 ± 0.62 | 63.2 ± 2.78 | 60.2 ± 1.67 |
| Glucan | 39.50 ± 0.41 | 52.49 ± 0.53 | 58.35 ± 0.3 | 60.55 ± 0.54 | 37.45 ± 0.6 | 49.52 ± 0.59 | 54.25 ± 0.12 | 56.62 ± 1.19 | 38.11 ± 0.1 | 53.60 ± 1.0 | 56.74 ± 1.31 | 60.07 ± 1.31 |
| Xylan | 25.21 ± 0.13 | 7.92 ± 1.35 | 4.88 ± 0.41 | 1.72 ± 0.09 | 24.81 ± 0.4 | 10.13 ± 0.66 | 6.62 ± 0.22 | 1.61 ± 0.08 | 24.21 ± 0.2 | 8.88 ± 0.19 | 4.53 ± 0.44 | 1.73 ± 0.24 |
| Arabinan | 1.23 ± 0.38 | 0.21 ± 0.04 | 0.21 ± 0.02 | 0.00 ± 0.00 | 2.73 ± 0.1 | 0.69 ± 0.06 | 0.36 ± 0.04 | 0.00 ± 0.00 | 1.48 ± 0.24 | 0.39 ± 0.05 | 0.28 ± 0.06 | 0.00 ± 0.00 |
| Acetyl | 3.43 ± 0.04 | 2.06 ± 0.37 | 1.24 ± 0.24 | 0.39 ± 0.01 | 2.21 ± 0.06 | 2.07 ± 0.09 | 1.23 ± 0.11 | 0.29 ± 0.03 | 4.32 ± 0.18 | 2.27 ± 0.05 | 1.43 ± 0.19 | 0.73 ± 0.08 |
| Lignin | 19.35 ± 0.06 | 29.41 ± 0.72 | 29.51 ± 0.4 | 33.29 ± 1.58 | 16.17 ± 0.8 | 25.78 ± 1.82 | 27.30 ± 0.34 | 30.64 ± 1.20 | 19.5 ± 0.59 | 28.5± 2.00 | 28.3 ± 0.29 | 32.6 ± 0.80 |
| Ash | 2.89 ± 0.65 | N. D | N.D | N.D | 7.34 ± 0.21 | N.D | N.D | N.D | 5.21 ± 0.71 | N.D | N.D | N.D |
| Extractives | 6.02 ± 0.42 | N. D | N.D | N.D | 12.07 ± 1.5 | N.D | N.D | N.D | 10.32 ± 0.4 | N.D | N.D | N.D |
| Water soluble sugars |  |  |  |  |  |  |  |  |  |  |  |  |
| Sucrose + fructose | 0.41 ± 0.01 | - | - | - | 1.02 ± 0.02 | - | - | - | 0.70 ± 0.02 | - | - | - |
| glucose + G-OS | 0.33 ± 0.01 | - | - | - | 0.44 ± 0.01 | - | - | - | 0.36 ± 0.01 | - | - | - |
| xylose + X-OS | 0.37 ± 0.01 | - | - | - | 0.40 ± 0.01 | - | - | - | 0.36 ± 0.02 | - | - | - |
| **Component in Liquor + WSS (g/100g RDM)** | | | | | | | | | | | | |
| Monomeric Glucose | - | 0.02 ± 0.00 | 0.64 ± 0.08 | 0.77 ± 0.06 | - | 0.04 ± 0.01 | 0.39 ± 0.03 | 0.45 ± 0.04 | - | 0.03 ± 0.00 | 0.34 ± 0.05 | 0.47 ± 0.05 |
| G-OS | - | 1.33 ± 0.04 | 1.74 ± 0.15 | 1.10 ± 0.15 | - | 1.42 ± 0.08 | 2.10 ± 0.36 | 1.43 ± 0.08 | - | 1.24 ± 0.05 | 2.05 ± 0.05 | 1.23 ± 0.05 |
| Monomeric Xylose | - | 1.69 ± 0.03 | 6.74 ± 0.47 | 2.74 ± 0.17 | - | 0.99 ± 0.04 | 5.01 ± 0.11 | 1.73 ± 0.05 | - | 1.52 ± 0.09 | 6.09 ± 0.60 | 2.49 ± 0.06 |
| X-OS | - | 15.22 ± 0.54 | 7.49 ± 0.62 | 2.04 ± 0.13 | - | 15.78 ± 0.20 | 12.60 ± 1.29 | 2.78 ± 0.21 | - | 15.27 ± 0.8 | 11.94 ± 0.94 | 2.10 ± 0.14 |
| Monomeric | - | 0.60 ± 0.07 | 0.71 ± 0.05 | 0.19 ± 0.11 | - | 1.00 ± 0.05 | 0.84 ± 0.09 | 0.15 ± 0.03 | - | 0.92 ± 0.05 | 0.75 ± 0.08 | 0.2 ± 0.01 |
| A-OS | - | 0.19 ± 0.04 | 0.21 ± 0.05 | 0.10 ± 0.08 | - | 0.75 ± 0.14 | 0.54 ± 0.28 | 0.10 ± 0.02 | - | 0.64 ± 0.15 | 0.42 ± 0.16 | 0.15 ± 0.07 |
| Furfural | - | 0.17 ± 0.00 | 0.51 ± 0.10 | 0.96 ± 0.03 | - | 0.20 ± 0.00 | 0.26 ± 0.04 | 0.80 ± 0.00 | - | 0.18 ± 0.00 | 0.32 ± 0.03 | 0.93 ± 0.01 |
| 5-HMF | - | 0.03 ± 0.00 | 0.15 ± 0.02 | 0.26 ± 0.01 | - | 0.03 ± 0.01 | 0.08 ± 0.009 | 0.30 ± 0.03 | - | 0.03 ± 0.01 | 0.07 ± 0.01 | 0.22 ± 0.00 |
| Acetic Acid | - | 2.78 ± 0.10 | 3.36 ± 0.17 | 3.68 ± 0.14 | - | 1.79 ± 0.06 | 2.13 ± 0.09 | 2.60 ± 0.04 | - | 2.38 ± 0.01 | 2.82 ± 0.06 | 3.37 ± 0.04 |
| Formic Acid | - | 0.20 ± 0.05 | 0.56 ± 0.05 | 0.66 ± 0.01 | - | 0.24 ± 0.01 | 0.54 ± 0.04 | 0.88 ± 0.01 | - | 0.28± 0.01 | 0.49 ± 0.06 | 0.52 ± 0.01 |
| pH | - | 3.60 ± 0.03 | 3.08 ± 0.03 | 3.05 ± 0.03 | - | 3.90 ± 0.01 | 3.63 ± 0.03 | 3.26 ± 0.01 | - | 3.76 ± 0.01 | 3.43 ± 0.00 | 3.17 ± 0.01 |
| StEx – Steam explosion, LS – low severity, MS – Mild or Intermediate severity, HS – High severity; G-OS – glucoligosaccharide; XOS – xyloligosaccharide; WIS – water insoluble solids; WSS – water soluble solids | | | | | | | | | | | | |

| **Pretreatment Conditions** | **Bagasse** | | | | **Trash** | | | | **Bagasse + Trash Mixture** | | | |
| --- | --- | --- | --- | --- | --- | --- | --- | --- | --- | --- | --- | --- |
|  | **Raw** | **LS - StEx** | **MS-StEx** | **HS - StEx** | **Raw** | **LS - StEx** | **MS-StEx** | **HS - StEx** | **Raw** | **LS - StEx** | **MS-StEx** | **HS - StEx** |
| **Major phenolic compounds in liquor (mg/L)** | | | | | | | | | | | | |
| Vanillic Acid | **-** | 1.34 | 8.08 | 16.3 | - | 0.92 | 11.19 | 17.98 | - | 0.88 | 11.20 | 16.31 |
| Vanillin | **-** | 0.38 | 57.2 | 146.6 | - | 0.16 | 63.48 | 84.8 | - | 0.25 | 50.34 | 100.6 |
| Syringic Acid | **-** | 0.61 | 174.4 | 200.2 | - | 0.37 | 121.8 | 128.75 | - | 0.72 | 151.0 | 161.0 |
| Syringaldehyde | **-** | 0.00 | 21.2 | 25.2 | - | 0.00 | 13.60 | 14.9 | - | 0.00 | 14.31 | 14.9 |
| p-Coumaric Acid | **-** | 0.89 | 26.3 | 99.7 | - | 0.14 | 16.10 | 47.14 | - | 0.31 | 20.78 | 63.11 |
| Ferulic Acid | **-** | 0.54 | 6.99 | 53.5 | - | 0.00 | 6.47 | 23.52 | - | 0.22 | 6.77 | 34.4 |
| Coniferyl Adlehyde | **-** | 0.00 | 4.07 | 8.66 | - | 0.00 | 4.46 | 5.14 | - | 0.00 | 4.35 | 8.11 |
| 3,4-Dihydrobenzoic acid | **-** | 0.00 | 7.53 | 18.22 | - | 0.00 | 10.33 | 17.09 | - | 0.00 | 7.01 | 17.53 |
| StEx – Steam explosion, LS – low severity, MS – Mild or Intermediate severity, HS – High severity; G-OS – glucoligosaccharide; XOS – xyloligosaccharide; WIS – water insoluble solids; WSS – water soluble solids; | | | | | | | | | | | | |
