# Supplementary material for: Ethanol production potential from AFEX™ and steam-exploded sugarcane residues for sugarcane biorefineries
Source: Biotechnol Biofuels. 2018 May 4;11:127. doi: 10.1186/s13068-018-1130-z (PMC5934847; doi:10.1186/s13068-018-1130-z)
Supplement: Supplementary file 2 — Additional file 2: Table S2. AFEX™ -bagasse pretreatment conditions used for evaluating the effect of pretreatment conditions on the monomeric glucose, xylose and combined sugar yield using a central composite design of experiments (DOE). [file 13068_2018_1130_MOESM2_ESM.docx]

**Additional File 2**

Table S2: AFEX^TM^ -bagasse pretreatment conditions used for evaluating the effect of pretreatment conditions on the monomeric glucose, xylose and combined sugar yield using a central composite design of experiments (DOE)

| Bagasse (DOE) - Residence Time Fixed at 30min | | | | | | | | |
| --- | --- | --- | --- | --- | --- | --- | --- | --- |
| RunOrder | PtType | Blocks | Temperature  (deg C) | NH_3_ Loading  (g:g DM) | H_2_O Loading  (g:g DM) | Glucose  Yield (%) | Xylose  Yield (%) | Combined Sugar  Yield (%) |
| 1 | -1 | 1 | 120.00 | 1.00 | 0.26 | 29.61 | 30.5 | 29.96 |
| 2 | 1 | 1 | 140.00 | 1.50 | 0.80 | 82.25 | 79.000 | 80.98 |
| 3 | 1 | 1 | 100.00 | 1.50 | 0.80 | 57.21 | 75.17 | 64.21 |
| 4 | 1 | 1 | 100.00 | 1.50 | 0.40 | 39.56 | 47.15 | 42.52 |
| 5 | -1 | 1 | 120.00 | 0.16 | 0.60 | 50.4 | 54.69 | 52.07 |
| 6 | -1 | 1 | 86.36 | 1.00 | 0.60 | 48.57 | 62.68 | 54.07 |
| 7 | -1 | 1 | 120.00 | 1.84 | 0.60 | 79.62 | 86.81 | 82.42 |
| 8 | -1 | 1 | 153.64 | 1.00 | 0.60 | 75.37 | 73.55 | 74.66 |
| 9 | 1 | 1 | 100.00 | 0.50 | 0.40 | 41.02 | 52.15 | 45.36 |
| 10 | 1 | 1 | 140.00 | 1.50 | 0.40 | 72.86 | 76.01 | 74.09 |
| 11 | 1 | 1 | 100.00 | 0.50 | 0.80 | 34.43 | 41.62 | 37.23 |
| 12 | 0 | 1 | 120.00 | 1.00 | 0.60 | 69.33 | 72.91 | 70.72 |
| 13 | 1 | 1 | 140.00 | 0.50 | 0.40 | 51.42 | 53.94 | 52.40 |
| 14 | -1 | 1 | 120.00 | 1.00 | 0.94 | 48.6 | 52.52 | 50.13 |
| 15 | 0 | 1 | 120.00 | 1.00 | 0.60 | 71.42 | 73.54 | 72.25 |
| 16 | 0 | 1 | 120.00 | 1.00 | 0.60 | 70.5 | 73.16 | 71.54 |
| 17 | 1 | 1 | 140.00 | 0.50 | 0.80 | 47.68 | 51.51 | 49.17 |
| 18 | 1 | 2 | 110.00 | 1.00 | 0.80 | 56.06 | 69.08 | 61.13 |
| 19 | 1 | 2 | 130.00 | 1.00 | 0.60 | 72.8 | 74.6 | 73.50 |
| 20 | -1 | 2 | 100.00 | 1.00 | 0.40 | 44.55 | 51.97 | 47.44 |
| 21 | 1 | 2 | 140.00 | 1.20 | 0.60 | 82.5 | 79.5 | 81.33 |
| 22 | 1 | 2 | 140.00 | 1.00 | 0.60 | 78.5 | 79.5 | 78.89 |

| Cane Leaf Matter (DOE) - Residence Time Fixed at 30min | | | | | | | | |
| --- | --- | --- | --- | --- | --- | --- | --- | --- |
| RunOrder | PtType | Blocks | Temperature  (deg C) | NH_3_ Loading  (g:g DM) | H2O Loading  (g:g DM) | Glucose  Yield (%) | Xylose  Yield (%) | Combined Sugar  Yield (%) |
| 1 | 1 | 1 | 140.00 | 1.50 | 0.40 | 83.24 | 69.55 | 78.05 |
| 2 | 0 | 1 | 120.00 | 1.00 | 0.60 | 81.77 | 70.28 | 77.47 |
| 3 | 0 | 1 | 120.00 | 1.00 | 0.60 | 80.84 | 69.61 | 76.65 |
| 4 | 1 | 1 | 140.00 | 0.50 | 0.40 | 70.47 | 59.14 | 66.24 |
| 5 | -1 | 1 | 86.36 | 1.00 | 0.60 | 71.33 | 63.00 | 68.31 |
| 6 | 1 | 1 | 100.00 | 1.50 | 0.80 | 80.29 | 71.54 | 77.10 |
| 7 | 1 | 1 | 140.00 | 0.50 | 0.80 | 77.07 | 65.52 | 72.74 |
| 8 | 1 | 1 | 140.00 | 1.50 | 0.80 | 88.79 | 73.43 | 82.93 |
| 9 | 0 | 1 | 120.00 | 1.00 | 0.60 | 80.81 | 69.18 | 76.46 |
| 10 | -1 | 1 | 120.00 | 0.16 | 0.60 | 66.64 | 57.85 | 63.43 |
| 11 | -1 | 1 | 120.00 | 1.00 | 0.26 | 78.62 | 68.31 | 74.80 |
| 12 | -1 | 1 | 120.00 | 1.84 | 0.60 | 89.66 | 73.77 | 83.58 |
| 13 | 1 | 1 | 100.00 | 1.50 | 0.40 | 80.09 | 70.99 | 76.42 |
| 14 | 1 | 1 | 100.00 | 0.50 | 0.40 | 67.02 | 59.29 | 63.90 |
| 15 | 0 | 1 | 120.00 | 1.00 | 0.60 | 80.15 | 68.77 | 75.55 |
| 16 | 0 | 1 | 120.00 | 1.00 | 0.60 | 81.48 | 69.58 | 77.36 |
| 17 | -1 | 1 | 153.64 | 1.00 | 0.60 | 80.56 | 66.56 | 75.25 |
| 18 | 1 | 1 | 100.00 | 0.50 | 0.80 | 68.76 | 60.74 | 65.52 |
| 19 | -1 | 1 | 120.00 | 1.00 | 0.94 | 74.76 | 67.61 | 72.23 |
| 20 | 0 | 1 | 120.00 | 1.00 | 0.60 | 78.85 | 68.46 | 74.66 |
| 21 | 1 | 2 | 110.00 | 1.00 | 0.80 | 77.11 | 66.51 | 73.52 |
| 22 | 1 | 2 | 130.00 | 1.00 | 0.60 | 80.59 | 64.94 | 76.16 |
| 23 | -1 | 2 | 100.00 | 1.00 | 0.40 | 73.67 | 63.78 | 70.36 |
| 24 | 1 | 2 | 137.00 | 1.20 | 0.77 | 84.00 | 71.50 | 80.00 |
| 25 | 1 | 2 | 140.00 | 1.00 | 0.70 | 83.00 | 71.00 | 77.00 |

Table S3: AFEX^TM^ -CLM pretreatment conditions used for evaluating the effect of pretreatment conditions on the monomeric glucose, xylose and combined sugar yield using a central composite design of experiments (DOE)
