# Supplementary material for: Ethanol production potential from AFEX™ and steam-exploded sugarcane residues for sugarcane biorefineries
Source: Biotechnol Biofuels. 2018 May 4;11:127. doi: 10.1186/s13068-018-1130-z (PMC5934847; doi:10.1186/s13068-018-1130-z)
Supplement: Supplementary file 3 — Additional file 3: Fig. S1. Design of experiments results for evaluating the effect of AFEX™ pretreatment conditions on the monomeric combined sugar yield from sugarcane bagasse and CLM. [file 13068_2018_1130_MOESM3_ESM.docx]

**Additional File 3**

Fig. S1-A: Contour plots illustrating the effect of temperature, ammonia loading and water loading on the combined monomeric sugar (glucose and xylose) yield for sugarcane bagasse and cane leaf matter.

**b. AFEX^TM^-Bagasse: Regression coefficients and *p*-values**

**a. AFEX^TM^-Bagasse: Residual plots for combined sugar yield**

**d. AFEX^TM^-CLM: Regression coefficients and *p*-values**

**c. AFEX^TM^-CLM: Residual plots for combined sugar yield**

Fig. S1-B: The statistical residual plots and regression coefficients used to validate ANOVA assumptions in evaluating the effect of AFEX^TM^ pretreatment conditions on the monomeric combined sugar yield from sugarcane bagasse and CLM. Abbreviations: S - Standard Error of the Regression, PRESS - Prediction Sum of Squares.
