# Supplementary material for: Ethanol production potential from AFEX™ and steam-exploded sugarcane residues for sugarcane biorefineries
Source: Biotechnol Biofuels. 2018 May 4;11:127. doi: 10.1186/s13068-018-1130-z (PMC5934847; doi:10.1186/s13068-018-1130-z)
Supplement: Supplementary file 4 — Additional file 4: Fig. S2. Profiling the effect of ammonia loading and temperature on the combined glucose and xylose yields for AFEX™-treated bagasse and cane leaf matter after 1% glucan loading enzymatic hydrolysis with 15 mg protein g−1 glucan. [file 13068_2018_1130_MOESM4_ESM.docx]

**Additional File 4**

**Fig. S2:** Profiling the effect of ammonia loading and temperature on the combined glucose and xylose yields for AFEX^TM^-treated bagasse and cane leaf matter after 1% glucan loading enzymatic hydrolysis with 15mg protein per gram glucan. Pretreatment water loading and residence time were fixed at 0.65 g H_2_O per gram DM and 30 minutes, respectively.

**Region of**

**Model validity**

**Region of**

**Bagasse-Trash**

**Co-pretreatment**
