# Supplementary material for: Ethanol production potential from AFEX™ and steam-exploded sugarcane residues for sugarcane biorefineries
Source: Biotechnol Biofuels. 2018 May 4;11:127. doi: 10.1186/s13068-018-1130-z (PMC5934847; doi:10.1186/s13068-018-1130-z)
Supplement: Supplementary file 5 — Additional file 5: Fig. S3. Statistical optimization of Cellic® CTec3, Cellic® HTec3, Pectinex Ultra-SP combinations for maximizing combined glucose and xylose yields from AFEXTM and Steam exploded sugarcane bagasse and CLM. [file 13068_2018_1130_MOESM5_ESM.docx]

**Additional File 5**

Fig. 3S-A: Quantification of the effect combinations of commercial enzyme mixtures consisting of Cellic CTec3, Cellic HTec3, and Pectinex Ultra SP on the combined sugar yield during 1% glucan loading enzymatic hydrolysis performed at 50 °C, 250 rpm for 72 hrs. The total enzyme dosage was fixed at 15mg/g glucan in pretreated biomass A – Contour profiles of the effect of commercial enzyme mixtures on the combined sugar yields, B – Composition of the optimized cocktail mixtures by ratio and the corresponding glucose, xylose and combined sugar yield.

**A**

**B**

Fig. 3B: The statistical residual plots and regression coefficients (coded) used to validate ANOVA assumptions in evaluating the effect of commercial enzyme cocktail mixtures on the monomeric combined sugar yield from AFEX^TM^-treated sugarcane bagasse and CLM. Abbreviations: S - Standard Error of the Regression, PRESS - Prediction Sum of Squares.

**a. AFEX^TM^-Bagasse: Residual plots for Enzyme Optimization**

**e. StEx-Bagasse: Residual plots for Enzyme Optimization**

**e. StEx-Bagasse: Residual plots for Enzyme Optimization**

**e. StEx-Bagasse: Residual plots for Enzyme Optimization**

**b. AFEX^TM^-CLM: Residual plots for Enzyme Optimization**

**e. StEx-Bagasse: Residual plots for Enzyme Optimization**

**e. StEx-Bagasse: Residual plots for Enzyme Optimization**

**e. StEx-Bagasse: Residual plots for Enzyme Optimization**

**c. AFEX^TM^-Bagasse: Regression coefficients and *p*-values**

**f. AFEX^TM^-Bagasse: Regression coefficients and *p*-values**

**f. AFEX^TM^-Bagasse: Regression coefficients and *p*-values**

**f. AFEX^TM^-Bagasse: Regression coefficients and *p*-values**

**c. AFEX^TM^-CLM: Regression coefficients and *p*-values**

**f. AFEX^TM^-Bagasse: Regression coefficients and *p*-values**

**f. AFEX^TM^-Bagasse: Regression coefficients and *p*-values**

**f. AFEX^TM^-Bagasse: Regression coefficients and *p*-values**

Fig. 3C: The statistical residual plots and regression coefficients (coded) used to validate ANOVA assumptions in evaluating the effect of commercial enzyme cocktail mixtures on the monomeric combined sugar yield from StEx-treated sugarcane bagasse and CLM. Abbreviations: S - Standard Error of the Regression, PRESS - Prediction Sum of Squares.

**f. StEx-Bagasse: Regression coefficients and *p*-values**

**f. AFEX^TM^-Bagasse: Regression coefficients and *p*-values**

**f. AFEX^TM^-Bagasse: Regression coefficients and *p*-values**

**f. AFEX^TM^-Bagasse: Regression coefficients and *p*-values**

**e. StEx-Bagasse: Residual plots for Enzyme Optimization**

**e. StEx-Bagasse: Residual plots for Enzyme Optimization**

**e. StEx-Bagasse: Residual plots for Enzyme Optimization**

**e. StEx-Bagasse: Residual plots for Enzyme Optimization**

**h. StEx-CLM: Regression coefficients and *p*-values**

**h. AFEX^TM^-CLM: Regression coefficients and *p*-values**

**h. AFEX^TM^-CLM: Regression coefficients and *p*-values**

**h. AFEX^TM^-CLM: Regression coefficients and *p*-values**

**g. StEx-CLM: Residual plots for Enzyme Optimization**

**g. StEx-CLM: Residual plots for Enzyme Optimization**

**g. StEx-CLM: Residual plots for Enzyme Optimization**

**g. StEx-CLM: Residual plots for Enzyme Optimization**
