# Supplementary material for: Ethanol production potential from AFEX™ and steam-exploded sugarcane residues for sugarcane biorefineries
Source: Biotechnol Biofuels. 2018 May 4;11:127. doi: 10.1186/s13068-018-1130-z (PMC5934847; doi:10.1186/s13068-018-1130-z)
Supplement: Supplementary file 6 — Additional file 6: Fig. S4. Material balances during pretreatment, washing, hydrolysis and fermentation for Processes I–IV. [file 13068_2018_1130_MOESM6_ESM.docx]

**Additional File 6**

Figure S4: Material balances during pretreatment, washing, hydrolysis and fermentation for Processes I – IV. A – AFEX-bagasse (Process I), B – StEx-bagasse (washed) (Process II), C – StEx-bagasse (unwashed) (Process III), D – StEx-bagasse (whole slurry) (Process IV), E – AFEX-CLM (Process I), F – StEx-CLM (washed) (Process II), G – StEx (unwashed) (Process III), H – StEx (whole slurry) (Process IV). Abbreviations: *WIS* – water insoluble solids, *WSS* – water soluble solids.
